# The Antiviral Compound PSP Inhibits HIV-1 Entry via PKR-Dependent Activation in Monocytic Cells

**DOI:** 10.3390/v15030804

**Published:** 2023-03-22

**Authors:** Eduardo Alvarez-Rivera, Madeline Rodríguez-Valentín, Nawal M. Boukli

**Affiliations:** 1 Biomedical Proteomics Facility, Department of Microbiology and Immunology, Universidad Central del Caribe School of Medicine, Bayamόn, PR 00960, USA; 2 Department of Microbiology, Houston Lee Moffitt Cancer Center and Research Institute, Tampa, FL 33612, USA

**Keywords:** PSP, HIV, proteomics, cofilin-1, PKR, IRE1α, UPR

## Abstract

Actin depolymerization factor (ADF) cofilin-1 is a key cytoskeleton component that serves to lessen cortical actin. HIV-1 manipulates cofilin-1 regulation as a pre- and post-entry requisite. Disruption of ADF signaling is associated with denial of entry. The unfolded protein response (UPR) marker Inositol-Requiring Enzyme-1α (IRE1α) and interferon-induced protein (IFN-IP) double-stranded RNA- activated protein kinase (PKR) are reported to overlap with actin components. In our published findings, *Coriolus versicolor* bioactive extract polysaccharide peptide (PSP) has demonstrated anti-HIV replicative properties in THP1 monocytic cells. However, its involvement towards viral infectivity has not been elucidated before. In the present study, we examined the roles of PKR and IRE1α in cofilin-1 phosphorylation and its HIV-1 restrictive roles in THP1. HIV-1 p24 antigen was measured through infected supernatant to determine PSP’s restrictive potential. Quantitative proteomics was performed to analyze cytoskeletal and UPR regulators. PKR, IRE1α, and cofilin-1 biomarkers were measured through immunoblots. Validation of key proteome markers was done through RT-qPCR. PKR/IRE1α inhibitors were used to validate viral entry and cofilin-1 phosphorylation through Western blots. Our findings show that PSP treatment before infection leads to an overall lower infectivity. Additionally, PKR and IRE1α show to be key regulators in cofilin-1 phosphorylation and viral restriction.

## 1. Introduction

The amount of globally infected individuals (38.4 million as of 2021) has surpassed those who have access to antiretroviral therapy (28.7 million in 2021) according to the statistics of the World Health Organization (WHO) and the United Nations Programme on HIV/AIDS. The corresponding amount has been slowly increasing throughout the years [1,2]. This renders at least 25% of the HIV-1 infected worldwide population without access to highly active antiretroviral treatment (HAART). While HAART is used to counteract the devastating effects of HIV-1, it remains an expensive solution [3,4]. Due to these documented facts, new treatment therapies co-working with HAART are required to combat HIV-1, as well as to ensure new preventative methods.

The success of traditional therapies by the 2015 Nobel Prize for an antimalarial treatment from Chinese herbs has renewed the interest in developing new drugs with immunomodulatory properties [5]. To that effect, a body of research demonstrates examples of mushrooms that can be used as a great source of natural compounds [6]. It undertakes this task by increasing the immunomodulatory response described by ancient Chinese history [7]. It has been demonstrated by our research group that *Coriolus versicolor* polysaccharide peptide (PSP) possesses anti-HIV properties by lowering viral replication by an average of 61% in a toll-like receptor 4 (TLR4)-dependent manner [8].

**Figure 1:**
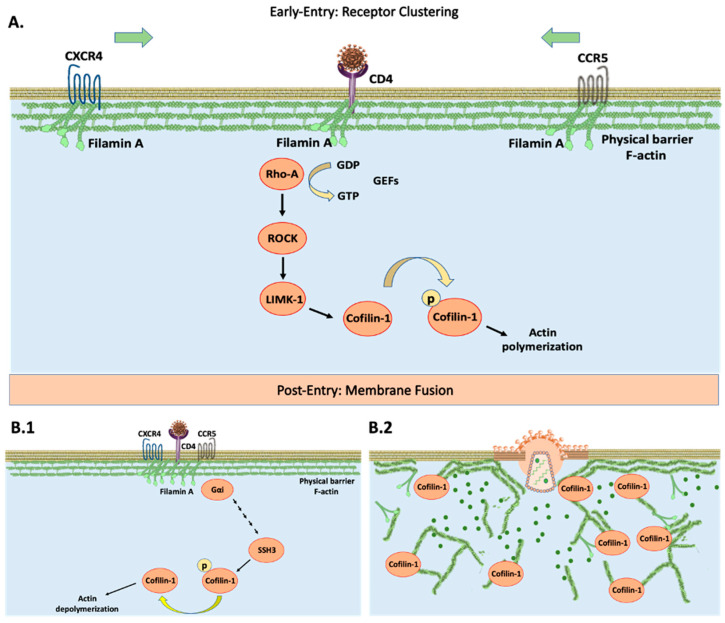
Diagram depicting HIV-1 entry during infection in CD4+ cells. (**A**) Early interaction between HIV-1 and CD4 receptor initiates activation of guanine nucleotide exchange factors (GEFs) and subsequently downstream signaling through RhoA/ROCK/LIMK-1. This results in phosphorylation and inactivation of cofilin-1. Actin dynamics will shift towards the polymerization state leading to CXCR4/CCR5 co-receptor clustering as the first requirement for viral entry. Filamin A serves as a linkage factor between CD4 and co-receptors. (**B.1**) HIV-1 associates with CXCR4/CCR5 in proximity and triggers downstream dephosphorylation and activation of cofilin-1. This process is carried out by G-protein coupled receptor signaling, specifically through Gα subunit-mediated phosphatases such as SSH3. (**B.2**) Cofilin-1 will begin the breakdown of actin filaments and subsequently lead to viral fusion as the final entry requirement.

HIV-1 targets CD4+ cells with CXCR4/CCR5 co-receptors such as monocytes/macrophages, dendritic, and T-helper cells [9,10,11,12]. HIV-1 interaction with CD4 remains an obstacle to viral entry. HIV-CD4/CXCR4/CCR5 activates Rho family GTPases to exchange GDP for GTP [13,14]. This leads to downstream phosphorylation of ROCK/LIMK-1 to inactivate actin-depolymerization factor (ADF) cofilin-1. This process is referred to as actin polymerization (Figure 1A). The strengthening/bundling of cortical actin filaments results in co-receptor clustering, increasing the probability of interaction towards the HIV-CD4 complex. At this stage, cytoskeleton remodeling effectively blocks entry by acting as a barrier [15] (Figure 1A). The HIV-CXCR4/CCR5 complex proceeds to recruit phosphatases to activate cofilin-1. This promotes the breakdown of actin filaments, effectively eliminating this barrier by actin depolymerization [16,17] (Figure 1B). Among the phosphatases regulating cofilin-1 activation are the slingshot homolog family: SSH1, SSH2, and SSH3 [18,19]. The exact signaling that HIV-1 uses to recruit these phosphatases remains elusive. HIV co-receptors are G-protein coupled receptors to which it holds influence towards the Gα subunit [20]. This affinity sets the stages of dephosphorylation and activation resulting in successful infection (Figure 1B). Interference with either cofilin-1 or its phosphorylated state from any of the pre-requisite steps leads to denial of entry [21,22].

The cytoskeleton contributes towards morphology, trafficking, migration, and adhesion [23,24,25], and ultimately acts as a deciding factor for HIV-1 entry [16,18,21,22,26,27,28]. Among its regulators, the actin binding protein (ABP) gelsolin is considered a strong influencer over actin dynamics. It holds the same function as cofilin-1, promoting the breakdown of actin filaments [29,30]. In contrast to its ADF counterpart, it is independent of HIV-1 signaling. The literature analysis shows that gelsolin overexpression interferes with the pre-fusion cascade, essentially blocking HIV-1 entry [31,32]. This unique property makes gelsolin a strong candidate as a restriction inducer. Although precise biological mechanisms remain elusive, evidence suggests that the endoplasmic reticulum (ER) plays a vital role in cytoskeleton regulation [33,34,35,36]. Aggregations of unfolded proteins due to cellular stress lead to an unfolded protein response (UPR). This characteristic is attributed to glucose-regulated protein 78 (GRP78) [37,38]. GRP78 up-regulation triggers ER stress by the Protein Kinase R-like ER kinase (PERK), activating transcription factor 6α (ATF6) and inositol requiring enzyme 1α (IRE1α) to decide cell fate. A short or prolonged UPR induces survival or apoptotic ER stress respectively [37,38,39], which overlaps with actin filaments and indirectly dictates the polymerization state [40,41,42,43]. Therefore, treatments that can interfere with HIV-1 depolymerization/polymerization status are pivotal for arresting entry at the pre- or post-fusion steps respectively.

The UPR aims at restoring cellular homeostasis following physiological stress exerted on the ER. This also invokes direct control of cytoskeleton re-arrangement in response to invading microorganisms [44,45]. In recent years, studies have surged to understand novel UPR overlapping signaling events. This survival mechanism has been shown to have an influence on the production of type I interferons (IFN) to ward off infections [46]. In a similar manner, it has been postulated that the interferon-induced protein (IFN-IP) double-stranded RNA-activated protein kinase (PKR) can interact directly with upstream regulators of cofilin-1 [47,48]. PKR has also been researched to be closely linked with the UPR [49]. Specifically, a study has shown the RNase activity of IRE1α can directly activate PKR downstream of TLR4 signaling [50]. The unique properties of PKR range from an immune response against viral infection to a survival regulator [51,52,53], but most importantly it has been linked to the phosphorylation of cofilin-1 (Ser3) [54].

The current study reveals, for the first time, the anti-HIV restrictive properties of PSP by influencing cofilin-dependent phosphorylation through PKR and IRE1α activation. Additionally, the identification of de-regulated cytoskeletal and UPR proteins were unveiled. Our findings establish a distinctive pattern of PSP before infection occurs as well as exerting control over cytoskeletal components. We show that the overexpression of PKR induced by PSP leads to a direct correlation to IRE1α and cofilin-1. These observations are based on our data that inhibition of either IRE1α or PKR rendered the cell vulnerable to infection by significantly increasing viral entry. These results provide the first insights into PSP’s restrictive roles through UPR, IFN-IP, and cytoskeletal markers.

## 2. Materials and Methods

### 2.1. Cell Culture

THP1-Blue-CD14 derives from the human monocytic THP1 cell line (InvivoGen, San Diego, CA, USA). These cells carry a reporter plasmid expressing a secreted embryonic alkaline phosphatase (SEAP) and express all TLRs; however, they only respond to ligands that are specific for TLR2, TLR1/2, TLR2/6, TLR4, TLR5, and TLR8. Cells were cultured in Roswell Park Memorial Institute (RPMI) 1640 medium (ATCC, Manassas, VA, USA), supplemented with 100 U/mL penicillin and 100 µg/mL streptomycin, in addition to 10% heat-inactivated fetal bovine serum (ATCC, Manassas, VA, USA). Cells were maintained in T-75 cm^2^ culturing flasks in a humidified incubator at 37 °C and 5% CO_2_.

### 2.2. PSP Extraction and Treatment

PSP supplement tablets consist of the following: 28% polysaccharide-to-peptide ratio with 60.23 mg/g beta-1,3/1,6-glucan (Mushroom Science, Eugene, OR, USA). To achieve the extracted working treatment, each tablet is diluted in hot/boiling water (approximately 90–100 °C) and centrifuged at 2060× *g* for 5 min. This cycle is repeated until the supernatant is free of insoluble residues. Ethanol 80% was used to separate the solution into two phases and PSP was collected from the light-brown layer. This was followed by washing with absolute ethanol, centrifugation, and drying with a refrigerator vapor trap (Thermo Fisher Scientific, Waltham, MA, USA) at −105 °C. The extracted treatment can be used directly or stored at −20 °C. A concentration of 200 μg/mL was used and defined after the assessment of cell activation and cytotoxicity for a 6 day period as previously published [8]. PSP was added twice during the allotted time (at days 0 and 3) for a period of 72 h, with fresh RPMI 1640 culture media to achieve the desired exposure concentration. Cells were maintained in T-75 cm^2^ cultured flasks, in a humidified incubator at 37 °C and 5% CO_2_.

### 2.3. HIV Stocks and Propagation

HIV-1ME46 (NIH AIDS Reagent Program, Bethesda, MD, USA) virus was propagated with ex vitro cell culture using peripheral blood mononuclear cells (PBMC) as recommended by the NIH HIV Reagent program’s protocol. Healthy PBMCs were extracted from blood donors using heparin tubes and subjected to centrifuging at 572× *g* for 12 min. Histopaque (Sigma-Aldrich, Saint Louis, MO, USA) was used to separate PBMCs from plasma and cell debris and subsequently washed with PBS 1X. Cells were cultured in RPMI 1640 (ATCC, Manassas, VA, USA) and incubated in T-75 cm^2^ flasks in a humidified incubator at 37 °C, 5% CO_2_. PBMCs were stimulated with 5 µg/mL of phytohemagglutinin and subjected to 200 µL of HIV-1ME46 after 3 days of stimulation. Infection lasted for a total period of 9 days with supplementation of fresh media every 3 days. PBMCs were centrifuged and HIV particles were measured by analyzing p24 antigens in cell culture supernatant through quantitative reverse transcription polymerase chain reaction (RT-qPCR).

### 2.4. HIV Infection

After reaching approximately 80% confluency, THP1-Blue-CD14 were centrifuged and re-suspended in RPMI 1640 culture medium. Cells were passaged into a T-75 cm^2^ flask with fresh media and supplemented with 8 µg/mL hexadimethrine bromide (Sigma-Aldrich, Saint Louis, MO, USA) to enhance the infection process. Acute infection was achieved by using a multiplicity of infection (MOI) of 1.4 from dual-tropism HIV-1ME46 (NIH AIDS Reagent Program, Bethesda, MD, USA) per 1 million healthy cells. HIV-1-infected-THP1 were incubated in a humidified atmosphere at 37 °C and 5% CO_2_ for a period of 24 h and subsequently washed with fresh culture media. To guarantee equal infection among cells with or without PSP treatment, infection was carried out in the same flask and then divided into their respective groups.

### 2.5. Viral Load Analysis

After 6 days of continuous exposure to PSP, cells were cultured in 12-well plates (Thermo Fisher Scientific, Waltham, MA, USA) using an optimal density of 3 × 10^6^ cells. Subsequently, cells were acutely infected with HIV-1ME46 (NIH AIDS Reagents Program, Bethesda, MD, USA) with an MOI of 1.4 for a period of 24 h. Control group is composed of THP1 monocytic cells infected with HIV-1. Hexadimethrine bromide (Sigma-Aldrich, Saint Louis, MO, USA) at a concentration of 8 µg/mL was used for enhancement of infection. HIV particles were measured by analyzing HIV p24 antigens in cell culture supernatant after 24 h of infection through RT-qPCR using a COBAS 6800 system (Roche Diagnostics Corporation, Indianapolis, IN, USA). HIV-specific primers were supplemented in the HIV-1 Master Mix Reagent 2 kit (Roche Diagnostics Corporation, Indianapolis, IN, USA). Denaturalization, annealing, extension steps, and cycle numbers were carried out according to Roche Diagnostic’s pre-determined protocols. Viral load calculations were determined as the percentage of total infection used relative to treated and infected cells.

### 2.6. IRE1α and PKR Inhibition Assays

During inhibition assays, THP1-Blue-CD14 cells were subjected twice (days 0 and 3) to 5-h treatment of either 56.09 nM Imidazolo-oxindole PKR inhibitor C16 (Sigma-Aldrich, Saint Louis, MO, USA) or 221.8 nM IRE1α Inhibitor III, 4µ8C (Sigma-Aldrich, Saint Louis, MO, USA) prior to PSP addition and infection. Cells were later exposed twice to PSP (200 µg/mL) during the same time frame for a total of six days. Dimethyl sulfoxide (DMSO) at 0.5% was used as a drug diluent during experiments and represents the vehicle group.

### 2.7. Quantitative Proteomics Experiments by Isobaric Labeling Described Below

#### 2.7.1. Protein Extraction and Digestion

Cells were centrifuged at 321× *g* for 7 min, after 6 days of continuous PSP exposure (200 µg/mL). THP1-Blue-CD14 were lysed using a combination of ultrasonic and SDS lysis buffer composed of 2% SDS *w*/*v* and 250 mM NaCl. Additionally, PhosSTOP (Roche, Madison, WI, USA) phosphatase inhibitors, 2 mM sodium vanadate, an EDTA-free protease inhibitor cocktail (Promega, Madison, WI, USA), and 50 mM HEPES adjusted at pH 8.5 were added to the mixture for protein effective membrane lysis and protein preservation. The following were added for each lysate: 5 mM of dithiothreitol (DTT) and 14 mM of iodoacetamide in the dark for 30 min with the purpose to serve as a reducing and alkylation agent respectively. Proteins were extracted by methanol and chloroform precipitation. Subsequently, washes composed of ice-cold acetone were used. Each pellet was left to dry and afterwards resuspended in a combination of 8 M Urea and 50 mM HEPES (pH 8.5). Protein concentrations were measured using a bicinchoninic acid assay kit (Thermo Fisher Scientific, Waltham, MA, USA) and with a spectrophotometer using the Molecular Devices VersaMax Absorbance Microplate Reader (GMI Trusted Laboratory Solutions, Ramsey, MN, USA) prior to digestion through proteases. Samples were then diluted with 4 M Urea, and digested with LysC (Wako, Japan) in a 1:50 enzyme-to-protein ratio overnight. Further dilutions were carried out with 1.5 M Urea concentration the next day. To finalize the digestive process, Trypsin (Promega, Madison, WI, USA) was added to a ratio of 1:100 (enzyme to protein) for 6 h at 37 °C. Afterwards, 200 μL of 20% formic acid (FA) (adjusted to pH 2.0) was used to acidify the samples. Each was subjected to C18 solid-phase extraction (SPE) (Sep-Pak, Waters, Milford, MA, USA).

#### 2.7.2. Tandem Mass Tagging (TMT) Labeling

TMT isobaric labeling was performed using the 6-plex TMT kit (Thermo Fisher Scientific, Waltham, MA, USA). TMT reagents at a concentration of 0.8 mg were dissolved in 40 μL of dry acetonitrile (Micro BCA, Thermo Fisher Scientific, Waltham, MA, USA), and 10 μL were added for each 0.2 μg of peptides. These were further dissolved in 100 μL of 200 mM HEPES, pH 8.5. After 1 h of incubation at room temperature, the reaction was quenched by adding 8 μL of 5% hydroxylamine. Lastly, each labeled peptide was combined, acidified with 20 μL of 20% FA (pH∼2.0), and concentrated via C18 SPE on Sep-Pak cartridges (50 mg bed volume).

#### 2.7.3. Reverse-Phase High-Performance Liquid Chromatography (HPLC)

TMT labeled HIV-infected-THP1 (controls and PSP treated) extracted peptides were subjected to basic-pH reverse phase fractionation. Peptides were solubilized in buffer composed of 5% ACN, 50 mM ammonium bicarbonate adjusted to pH 8.0. These samples were stably separated through HPLC using an Agilent 300Extend-C18 column (Specification: 5 μm particles, 4.6 mm i.d. and 220 mm in length). Additionally, the flow rate of the samples was measured using an Agilent 1100 binary pump for HPLC. This was equipped with a degasser and a photodiode array detector (Thermo Fisher Scientific, Waltham, MA, USA), with a 45 min linear gradient ranging from 8–35% acetonitrile in 10 mM ammonium bicarbonate pH 8.0 (flow rate parameters: 0.8 mL/min). The peptide mixtures were separated into a total of 96 fractions as a result of this. Subsequently, said fractions were consolidated into two sets of samples in a checkerboard manner, acidified with 10 μL of 20% FA, and vacuum dried. Each sample was re-dissolved in 5% FA, desalted via StageTip, dried via vacuum centrifugation, and reconstituted for LC–MS/MS analysis.

#### 2.7.4. Triple Stage Mass Spectrometry (MS3)

During mass spectrometry (MS) experiments, all spectra were acquired on an Oribtrap Fusion (Thermo Fisher Scientific, Waltham, MA, USA) coupled to an Easy-nLC 1000 (Thermo Fisher Scientific, Waltham, MA, USA) ultrahigh pressure liquid chromatography (UHPLC) pump. Peptides were separated on a 100 μm inner diameter column composed of 0.5 cm of Magic C4 resin (5 μm, 100 Å, Michrom Bioresources, Auburn, CA, USA), followed by 25 cm of Sepax Technologies GP-C18 resin (1.8 μm, 120 Å, Newark, DE, USA) with a gradient ranging between 3–25% (ACN, 0.125% FA) in a time course of approximately 180 min.

For all experiments, the instrument was operated in the data-dependent mode. First-stage MS1 spectra were collected at a resolution of 120,000 using an automated gain control (AGC) target of 200,000 and a maximum injection time of 100 ms. A selection of the 10 most intense ions was used for the second stage MS2 approach. Precursors were filtered out according to relative charge state and monoisotopic peak assignments. These were later excluded using a dynamic window of 75 s ± 10 ppm and isolated with a quadrupole mass filter set to a width of 0.5 *m*/*z*.

During the second-stage MS2 spectra, the Orbitrap was operated at 60,000 resolutions, consisting of an AGC target of 50,000 and a maximum injection time of 250 ms. Precursors were fragmented by high-energy collision dissociation (HCD) at a normalized collision energy (NCE) of 37.5%.

Lastly, the MS3 approach was performed using the collected spectra during MS2 at an AGC of 4000, maximum injection time of 150 ms, and collision energy of 35%. The same Orbitrap parameters as for the MS2 method were used during MS3, with the exception that the HCD collision energy was increased to 55% to ensure maximal TMT reporter ion yield. The synchronous-precursor-selection (SPS) was enabled to include up to 3, 6, or 10 MS2 fragment ions in the MS3 scan.

#### 2.7.5. MS3 Data Processing

All data relating to thermo.“raw” files were converted into a readable file format (mz.XML) through a compilation of in-house software. This was also used to correct monoisotopic *m*/*z* measurements and incorrect peptide charge states that may have surged during the implementation of these methods. The distribution of MS2 spectra was performed using the SEQUEST algorithm. Each proteome experiment utilized the Human UniProt database to establish the accession number, gene name, and functions. For every experiment, reverse protein sequences were included for common contaminants such as human keratins. During SEQUEST analysis, a 50 ppm precursor ion tolerance was performed, while requiring each peptide’s N/C terminus to have trypsin protease specificity and allowing up to two missed cleavages. MS2 spectra assignment false discovery rate (FDR) of less than 1% was achieved by applying the target-decoy database search strategy.

#### 2.7.6. Determination of TMT Reporter Ion Intensities and Quantitative Data Analysis

For quantification, a 0.03 *m*/*z* (6-plex TMT) window centered on the theoretical *m*/*z* value of each reporter ion was queried for the nearest signal intensity. Reporter ions were adjusted to correct for the isotopic impurities of the different TMT reagents (manufacturer specifications). The signal-to-noise values for all peptides were summed within each TMT channel, where each was scaled according to the inter-channel differences to account for differences in sample handling. A total minimum sum of signal-to-noise values greater than 100 and isolation purity greater than 50% was required for each sample.

### 2.8. Western Blot

A concentration of 30 µg of protein samples extracted with RIPA buffer (Thermo Fisher Scientific, Waltham, MA, USA) was used for SDS-PAGE and transferred to a polyvinylidene difluoride membrane. A protease and phosphatase inhibitor cocktail (Thermo Fisher Scientific, Waltham, MA, USA) was used during the protein extraction. Protein concentrations were measured spectrophotometrically using the Molecular Devices VersaMax Absorbance Microplate Reader (GMI Trusted Laboratory Solutions, Ramsey, MN, USA). Bovine serum albumin (Fisher Scientific, Hampton, NH, USA) 5% in 1X TBST served as the blocking buffer for each membrane. Primary antibody incubation was done overnight at 4 °C in a shaker. The following primary antibodies were used at a 1:1000 dilution: cofilin-1, p-cofilin-1 (Ser 3), gelsolin, PKR, IRE1α, and GRP78, (Cell Signaling Technologies, Danvers, MA, USA), pPKR (Thr 446, Abcam, Cambridge, UK). Anti β-Actin (Cell Signaling Technologies, Danvers, MA, USA) was used as a loading control. Horseradish peroxidase (HRP)-conjugated secondary antibodies (Cell Signaling Technologies, Danvers, MA, USA) were used at a dilution of 1:10,000. Protein bands were visualized by chemiluminescence using the SuperSignal West Femto Maximum Sensitivity Substrate Kit (Thermo Fisher Scientific, Waltham, MA, USA). All images were analyzed with ImageJ image processing program version 1.52a (National Institute of Health, Bethesda, MD, USA).

### 2.9. Quantitative Reverse Transcription PCR

RNA was extracted from cell pellets using the RNeasy Plus Mini Kit (Qiagen, Hilden, Germany) following the manufacturer’s protocols. RNA quality and concentration were quantified spectrophotometrically with a NanoDrop 1000 spectrophotometer (Thermo Fisher Scientific, Waltham, MA, USA). cDNA was reverse transcribed from 1 µg of total RNA using the iScript cDNA synthesis kit (Bio-Rad, Hercules, CA, USA). Samples were then processed through RT-qPCR for identification of cofilin-1, gelsolin, and PKR genes by means of SYBR green assays (Bio-Rad, Hercules, CA, USA) using 500 nM of primers (Sigma-Aldrich, Saint Louis, MO, USA). Amplification was carried out in a Bio-Rad CFX96 Touch real time PCR detection system (Bio-Rad, Hercules, CA, USA) using the following parameters: 15 s at 95 °C, 1 min at the gene-specific annealing temperature, and 1 min at 72 °C for a total of 40 cycles. The gene-specific primers were as follows: cofilin-1 forward, 5′-GCTTCTTCTTGATGGCGTCCTTG-3′ and cofilin-1 reverse 5′-GTCACCTGTGGCTTTGCTGT-3′; gelsolin forward, 5′-CTACCCAGGATGAGGTCGCT-3′ and gelsolin reverse, 5′-GTGCCGCCCTTGTAGATGA-3′; PKR forward, 5′-TCCAACACTGGACTCCCTTC-3′; PKR reverse, 5′-TACTGGGGGCATATGGGTAA-3′. The gene expression level was defined as the threshold cycle number (CT). The mean fold changes in expression of the target genes were calculated using the comparative CT method (RU, 2ΔΔCT). All data were normalized through the quantity of RNA input of 18S forward, 5′-GGCCCTGTAATTGGAATGAGTC-3′ and 18S reverse, 5′-CCAAGATCCAACTACGAGCTT-3′, serving as the endogenous control and for normalization.

### 2.10. MTT Cell Viability Assay for IC50 Determination

The half inhibitory concentration (IC50) for Imidazolo-oxindole PKR inhibitor C16 (Sigma-Aldrich, Saint Louis, MO, USA) and IRE1α Inhibitor III, 4µ8C (Sigma-Aldrich, Saint Louis, MO, USA) were determined using Thiazolyl Blue Tetrazolium Bromide (Sigma-Aldrich, Saint Louis, MO, USA) MTT cell cytotoxicity assays. THP1-Blue-CD14 cells were seeded at a density of 1 × 10^4^ cells per well using a 96-well plate model in biological and technical triplicates for a period of 24 h. The next day, cells were exposed to either C16 or 4µ8C inhibitor at days 0 and 3 for a total of 6 days to simulate PSP treatments. The range of concentrations used for C16 and 4µ8C were 0–200 nM and 0–300 nM respectively. On the sixth day, 5 mg/mL of MTT was added to each sample using 1X PBS which served as the diluent as stated by Sigma-Aldrich protocols. After 4 h of incubation, the formazan crystals were dissolved using 100% DMSO. The absorption of the formazan solution was measured using the Molecular Devices VersaMax Absorbance Microplate Reader (GMI Trusted Laboratory Solutions, Ramsey, MN, USA) at a wavelength of 570 nm. Cell viability was calculated as the percentage of THP1-BLUE-CD14 treated cells relative to positive and negative controls. MTT IC50 results can be found in Figure S1.

### 2.11. Statistical Analysis

All data sets are expressed as mean ± S.E.M. for biological triplicates. Statistical analysis for in vitro studies was performed by using one way analysis of variance (ANOVA) with post hoc Tukey for multiple comparison groups or unpaired t-test as appropriate for each experiment, using GraphPad PRISM v9.5.0 (GraphPad Software, San Diego, CA, USA) statistical power software. A *p*-value of *p* ≤ 0.05 was considered as statistically significant.

## 3. Results

### 3.1. Isobaric TMT Labeling Quantitative Proteomics Profiling Revealed Differential Regulated Proteins Associated with Cytoskeletal and ER Stress Function Categories in THP1-Blue-CD14 Cells Treated with PSP

Untreated and PSP (200 µg/mL) treated (24 h) THP1 cells were used in the differential protein expression analysis. Relative high-throughput estimation of cellular protein abundances and quantitation have been achieved using stable isotope chemical labeling referred to as TMT labeling of proteins or peptides using MS. This quantitative proteomics approach has been widely used in our laboratory [55,56] for its high multiplexing capacity and deep proteome coverage. Proteins relating to cytoskeletal re-arrangement and ER stress UPR process with *p*-values ≤ 0.05 (*p*-value adjusted as false discovery rate) were deemed as statistically significant and selected for Supplementary Tables S1 and S2 respectively. The detailed information for accession number, gene symbol, number of spectra counts, protein name, average expression in control relating to treatment, fold change, and *p*-value are included in Tables S1 and S2. Due to the infeasibility to discuss all identified proteins in the MS data, the selection criteria are based on the significance of the fold change for each category of signaling markers.

A total of 111 proteins were associated with cytoskeletal functions and were identified as significantly expressed in PSP-treated cells. From these, 54 upregulated and 57 downregulated proteins make up the total. Among the differentially expressed proteins, the key factor of HIV entry involved in cofilin-1 activation, SSH3 was found to be downregulated (−1.46-fold). Interestingly, the interferon-induced, double-stranded RNA-activated protein kinase (EIF2AK2), which corresponds to PKR, is found significantly upregulated (2.44 fold). Actin-binding regulators tropomyosin and tropomodulin are identified as up-regulated (1.48 and 1.53 folds respectively). These two proteins need special mention since they are involved in sensing and modulating cofilin’s depolymerization activity [57,58,59]. In addition, seven guanine nucleotide exchange factors (GEFs) pertaining to the Rho, Ras, and Rab families (Table S1) involved in regulating the polymerization state of actin filaments are found de-regulated. An adapter and regulator of actin cytoskeletal, switch associated protein 70 (SWAP70), had a significant decrease in expression (−1.26). This marker has been proposed as a diagnostic marker for HIV-1 infection due to its presence in the membrane surface of HIV-positive cells [60]. Additionally, this protein has close ties with the GEF family, modulating their activity [61]. This correlates to the decrease in fold value of the Ras-specific guanine nucleotide release factor RalGPS2 (−1.43). Cytoskeletal and transport motor proteins such as myosin phosphatase Rho-interactin (MPRIP), unconventional myosin-XVIIIa (MYO18A), and tubulin-specific chaperone C (TBCC) have been listed in downregulated states: −1.24-, −1.28- and −1.24-fold respectively. Meanwhile, unconventional myosin-Ig (MYO1G) is upregulated by 1.34-fold. Phosphatidylinositol 3,4,5-triphosphate dependent Rac exchanger-1 (PREX1) is associated with the Rho family GTPases of Rac, Ral, and Rho. Additionally, it is a downstream cytoskeletal modulator of CXCR4 co-receptors relating to viral membrane fusion [35]. This protein is identified as significantly downregulated (−1.38) in the presence of PSP. This regulation correlates with its association with the Rho GTPase-activating protein 15 and 17 (−1.29 and −1.37-fold respectively). Among other HIV-1 entry factors, α-actinin was found to be downregulated (−1.20-fold). This protein has been known as a marker for virion fusion. [62]. Other facilitators of viral entry, such as microtubule-actin cross linking factor 1 (isoforms 1/2/3/5) are determined to be downregulated by −1.29-fold in treated cells. These cytoskeletal components are known for their internalization of HIV-1 capsid interactions during the early fusion steps [63].

Moreover, MS3 analysis has revealed 28 deregulated proteins (21 up- and seven downregulated) relating to UPR (Table S2). Among these, associated UPR and ER stress markers such as protein niban (FAM129A), ER degradation-enhancing alpha-mannosidase-like protein 1 (EDEM1), and heat shock protein 90-beta (HSP90AB1) were upregulated by 1.35-, 1.25- and 1.16-fold respectively. These markers are known for targeting translational regulation, misfolded proteins, and chaperone activity respectively [64,65,66]. In correlation with the UPR and EDEM1, F-box only protein 6 (FBXO6) is involved in the ER-associated degradation pathway for misfolded proteins. FBXO6 has also been associated with the inhibition of chronic ER stress, making it a suitable pro-survival marker [67]. FBOX06 has been significantly present in an upregulated state corresponding to 1.71-fold. PSP also extends its UPR signaling by positively modulating pro-survival chaperones. A clear indicator of this involves the upregulation (1.31-fold) of heat shock protein 105 kDa (HSPH1), which is involved in alleviating protein accumulation [68]. The effect of PSP also targets markers that possesses duality roles, such as heat-shock protein beta-1 (HSPB1). The overall levels of this marker were decreased by 1.14-fold. This protein has been associated as a cytoskeletal regulator and molecular chaperone [69]. Most notably, PSP treatment led to the significant upregulation of the ER survival chaperone protein disulfide isomerase (PDI) by a 1.17-fold change. Lastly, the UPR marker serine/threonine-protein kinase/endoribonuclease IRE1α (ERN1) is significantly overexpressed (1.41-fold).

### 3.2. PSP Treatment Leads to an Increase in Viral Restriction

**Figure 2:**
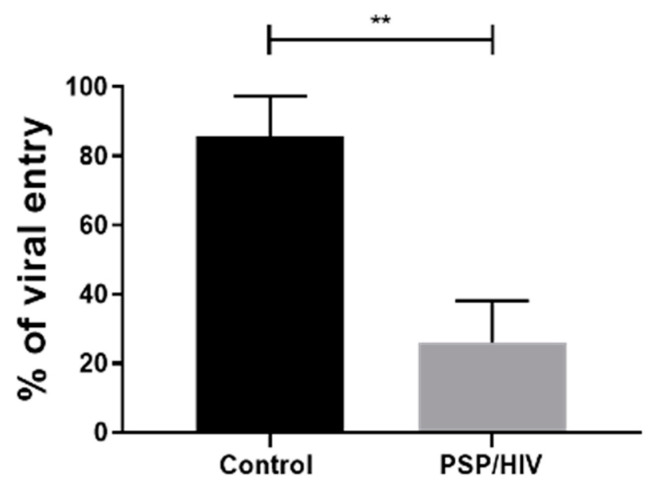
PSP lowers HIV entry in THP1 cells. Percentages of HIV entry in PSP-treated cells analyzed through viral load of HIV p24 antigen and compared with unpaired *t* test. Data are represented as mean ± SEM. Statistically significant difference (**), *p* < 0.01 is shown, *n* = 3.

Our research group has previously published a significant internal viral inhibition corresponding to 61% for PSP-treated HIV-infected THP1 and 35.99% PBMC [8]. To further widen the knowledge of these studies, the external antiviral effect of PSP was evaluated in THP1 cells. With the goal of determining PSP’s efficacy as an anti-HIV-1 restrictive agent, THP1 cells were supplemented twice with 200 µg/mL of PSP over a six-day period before succumbing to HIV-1. Infection was carried out for a total time lapse of 24 h on the sixth day relative to treated cells. Hexadimethrine bromide at a concentration of 5 µg/mL was added as an enhancer of infection and a cationic polymer which carries the effect of neutralizing charge repulsions between viral and cell surface membranes. HIV-1 entry was measured by analyzing the concentration of HIV-1 core p24 antigen through RT-qPCR in the cell culture supernatant. Viral load analysis revealed a significant reduction in HIV-mediated entry by an average of 26% relative to 85.67% in the control group, with a mean difference of 59.67 ± 9.735 (Figure 2). This result correlates to a restriction average of 74% in contribution to the effects of PSP.

### 3.3. Inhibition of PKR and IRE1α Are Associated with an Increase in HIV-1 Entry

**Figure 3:**
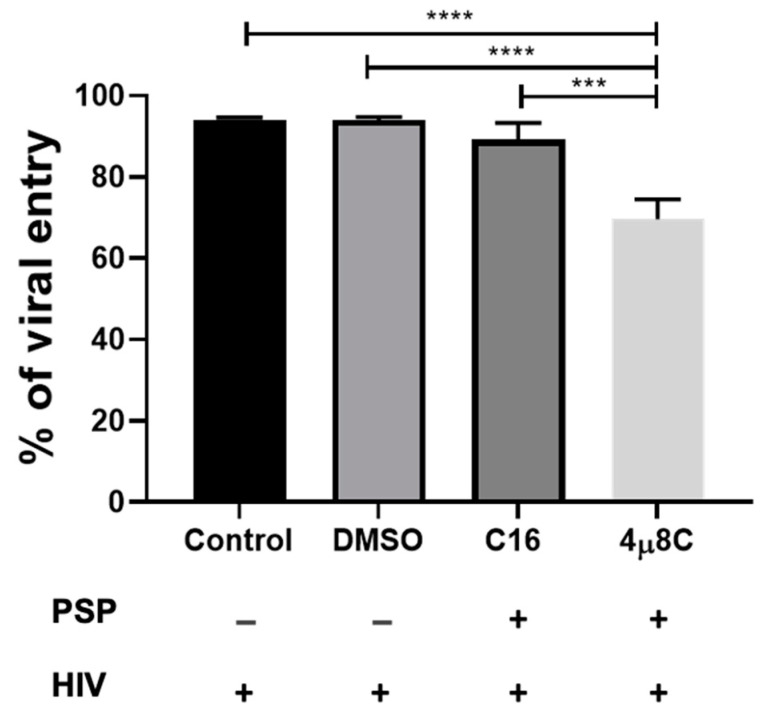
Inhibition of PKR and IRE1α activity facilitates HIV-1 entry. Percentages of HIV entry in PSP-treated cells supplemented with either 56.09 nM of C16-PKR or 221.8 nM of 4µ8C-IRE1α pharmaceutical blockers. Experiments were analyzed through viral loads of the HIV p24 antigen and compared with one-way ANOVA with Tukey multiple comparisons tests. Data are represented as mean ± SEM. Statistically significant difference (***), *p* < 0.001 and (****), *p* < 0.0001 are shown, *n* = 3.

Our data indicate that PSP has the potential to restrict HIV-1 entry as well as to induce the differential regulation of cytoskeletal and UPR proteins. To further investigate the signaling complex that influences this restrictive pattern, the potential roles of PKR and the UPR marker IRE1α in HIV-1 entry were studied by subjecting THP1 cells to C16-PKR and 4µ8C-IRE1α pharmaceutical inhibitors. We hypothesized that inhibition of either the activation of PKR or the endoribonuclease activity of IRE1α would affect viral entry into host immune cells. This is based on the overlapping signaling complex with downstream cytoskeletal effectors as seen in the literature research. Therefore, THP1 cells were treated twice with either 56.09 nM of C16 or 221.8 nM of 4µ8C for a period of 5 h prior to PSP treatment and HIV-1 infection. DMSO 0.5% was used as a vehicle control for both blockers. Viral load analysis has revealed no statistical difference in HIV-1 entry for C16, with approximately 89.13% relative to 94.1% for the infected control and 93.96% for the DMSO vehicle group (Figure 3). This correlates to a restrictive percentage of 10.87% for C16-treated PSP-induced cells. Compared to Figure 2, there was a relative increase in viral entry by approximately 63.13% in difference. Moreover, 4µ8C-treated PSP-induced cells showed approximately 69.56% of viral entry (24% restriction relative to the control and vehicle groups). The effects of 4µ8C can be translated to a 19.57% difference in comparison to the C16 blocker, contributing to a significant reduction in viral access (Figure 3). Taking into consideration the data in Figure 2, the inhibition of IRE1α endoribonuclease activity represents an approximate increment difference of 43.56% in viral entry. The results for both drugs show an inversely proportional HIV-1 entry and restriction taking place compared to Figure 2.

### 3.4. PSP Induces the Upregulation of Key Cytoskeletal, IFN-IP, and ER Stress Markers

**Figure 4:**
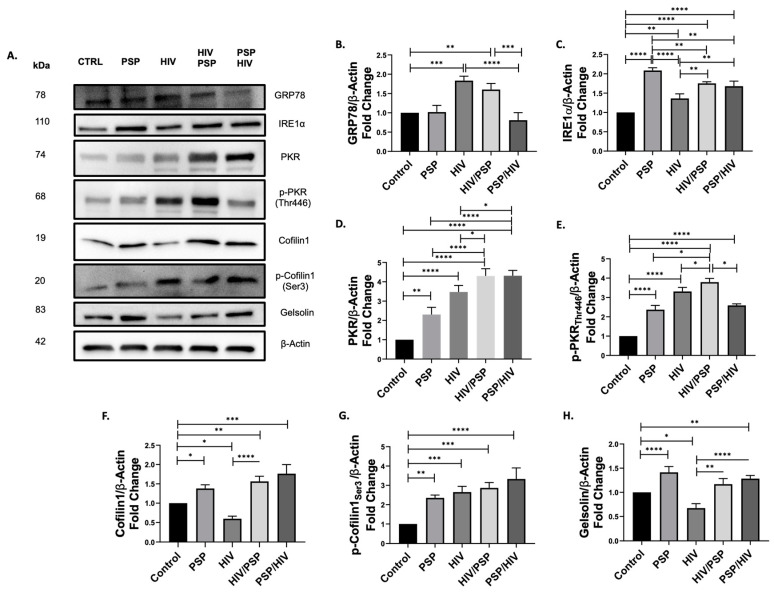
Western blot results related to UPR, IFN, and cytoskeletal biomarkers in PSP-treated THP1 monocytic cells. (**A**) Representative Western blot data for the protein expression levels of UPR: (**B**) GRP78; (**C**) IRE1α; IFN: (**D**) PKR; (**E**) p-PKR; ADF: (**F**) Cofilin1; (**G**) p-Cofilin1; and ABP: (**H**) Gelsolin signaling. β-Actin was used as a loading control and for normalization of data. Images were quantified using the ImageJ software (NIH, version 1.52a) by comparing the integrated density value with the control group. Mean ± SEM and significant difference (*), *p* ≤ 0.05, (**), *p* ≤ 0.01, (***), *p* ≤ 0.001, (****), *p* ≤ 0.0001 are shown and were determined using one-way ANOVA with Tukey multiple comparisons test, *n* = 3. All Western blot images can be found in Figure S2.

To confirm the regulative patterns obtained through quantitative proteomic analysis results (Tables S1 and S2), we explore the effects of PSP on proteins associated with overlapping cytoskeletal, IFN-IP signaling, and UPR complexes. The selective protein expression levels relating to cofilin-1, gelsolin, PKR, GRP78, and IRE1α were evaluated with Western blot (Figure 4A). Moreover, to grant further insight into PSP’s role in its protein regulation, we examined the effects of PSP before (PSP/HIV) and after (HIV/PSP) infection. Immunoblot data showed that there was no statistical difference for the master regulator of UPR and ER stress marker, GRP78, between PSP-treated cells and the control (Figure 4B). However, HIV infection resulted in an increase in protein expression levels for GRP78 of an average of 1.83-fold. A similar pattern was found in the HIV/PSP group with an increase of 1.60-fold. We observed that PSP lowered GRP78 expression in all instances where treatment was added alone or before infection, with no significance relative to control. The reverse effect was seen for HIV alone or after infection.

The expression patterns for IRE1α were upregulated in all instances for PSP and HIV infection. The highest statistical significance of a 2.08-fold change was seen for PSP alone in comparison to every sample group present (Figure 4C). HIV-1 infection demonstrated the lowest value of a 1.36-fold average relative to the control. In relation to its before and after infection counterparts, IRE1α expression was significantly higher, amounting to 1.75- and 1.68-fold respectively. There was no statistical difference reported relating to HIV/PSP vs. PSP/HIV samples.

Given that PKR has a history of interacting and overlapping with IRE1α and cofilin-1, we included this marker in our Western blot approaches as a marker of interest (Figure 4D). Results demonstrated that PSP has a strong influence on total PKR regulative pattern with a 2.30-fold change vs. the control. Similarly, PKR total expression levels increased in all groups, with the highest statistical significance in PSP treatment before and after infection (4.30- vs. 4.31-fold average respectively). In relation to these two experimental samples, there was no statistical difference seen between each other. Correspondingly, the activated and phosphorylated form of PKR at threonine 446 had a similar role to its total PKR counterpart, favoring upregulation in every group present (Figure 4E). PSP showed significantly higher activation by 3.79-fold expression in HIV/PSP relative to every group present. This data suggests an upregulation for both total and phosphorylated forms of PKR occurring in PSP-treated and infected cells.

ADF cofilin-1 and ABP gelsolin are strong influencers over HIV-1 entry, and it is for these reasons that they were of particular interest to reveal their regulative state in PSP-treated cells. A significant increase in cofilin-1 expression was seen in all instances where PSP was present (Figure 4F). In the present scenario, PSP/HIV had the highest fold value averaging a 1.76-fold difference before infection. Contrary to the previous results for the UPR and IFN-IP markers, there was a significant decrease average of a 0.59-fold change in total cofilin-1 vs. the control for HIV-1 infection. The deactivated/phosphorylated form of cofilin-1 had a similar pattern to the regulation of IRE1α and PKR. These show a tendency towards upregulation in every experimental situation (Figure 4G). Viral infection also demonstrated an increase of a 2.65-fold average of p-cofilin-1 compared to its total counterpart (Figure 4F). The highest reported fold difference of 3.33-fold was revealed before infection took place relative to the control. Lastly, immunoblot data for gelsolin revealed a slight increase in expression levels in all instances where PSP was present with a 1.41-fold difference as the highest in treatment only (Figure 4H). Equivalently to total cofilin-1 expression, viral infection received a significant decrease of 0.67-fold for cytosolic gelsolin. In summary, this data showed increased protein expression levels for key cytoskeletal, UPR, and IFN-IP markers.

### 3.5. Inhibition of PKR Modulates Cofilin-1 Phosphorylation Expression Patterns

**Figure 5:**
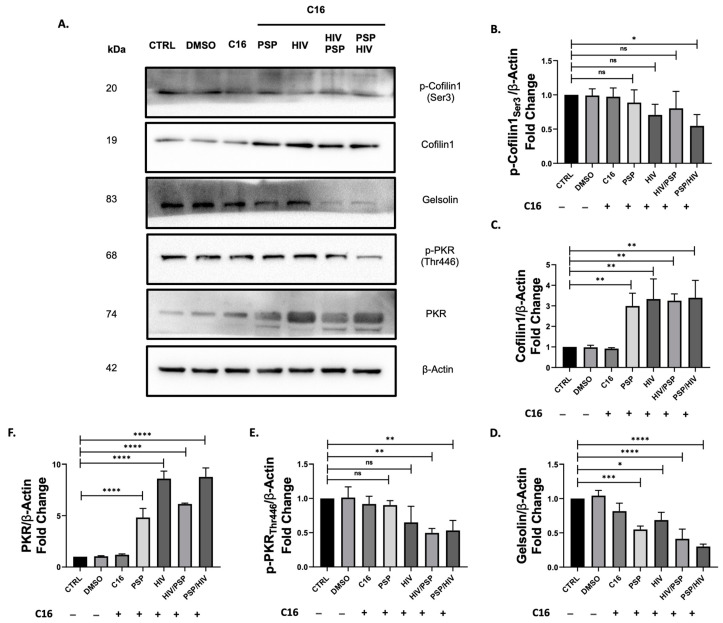
PKR inhibition decreases cofilin-1 phosphorylation and the expression levels relating to gelsolin. The activation of PKR was suppressed after 56.09 nM of C16 inhibitor in THP1 monocytic cells with or without PSP treatment. (**A**) Representative Western blot data for the protein expression levels of cytoskeletal: (**B**) pCofilin1; (**C**) Cofilin1; (**D**) Gelsolin; and IFN-IP: (**E**) pPKR; (**F**) PKR. β-Actin was used as a loading control and for normalization of data. Images were quantified using the ImageJ software (NIH, version 1.52a) by comparing the integrated density value with the control group. Mean ± SEM and significant difference (*), *p* ≤ 0.05, (**), *p* ≤ 0.01, (***), *p* ≤ 0.001, (****), *p* ≤ 0.0001 are shown and were determined using one-way ANOVA with Tukey multiple comparisons test, *n* = 3. All Western blot images can be found in Figure S2.

We previously showed that PSP has the capacity to upregulate both total and phosphorylated forms of cofilin-1 in addition to a slight increase in the ABP gelsolin. We also demonstrated that PSP favors upregulation towards the activated and de-activated states of PKR as well as IRE1α. Our viral load data showed that viral entry increases under a PKR-inhibited state. Based on these results, it is possible PKR is involved as an upstream regulator of cofilin-1-mediated phosphorylation, serving as an entryway key factor during HIV infection. Therefore, we aim to assess how cofilin-1 and gelsolin are affected by PKR inhibition. We treated THP1 monocytic cells with 56.09 nM of C16 pharmacological blocker at PKR threonine 443 and subjected the cells to PSP treatment and infection as previously done in our experimental methodologies. Immunoblots showed inverse results to what was observed in PSP-treated cells without any drug intervention. C16 implementation significantly decreased cofilin-1 phosphorylation similar to that of the control group (Figure 5B). PSP treatment before infection suffered the lowest downregulation of 0.54-fold expression vs. control. Interestingly, the total activated form of cofilin-1 resulted in a significant increase in fold expression for every experimental group present (Figure 5C). Taking into consideration the results from Figure 4F, every sample group demonstrated an increase of ± 2-fold with the infection group alone extending this by almost a 5-fold difference. In an opposite manner compared to p-cofilin-1, the highest expression value for its activated counterpart was seen with PSP treatment before infection. This expression amounts to an average of 3.39-fold expression compared to 0.54-fold in phosphorylated cofilin-1. Similar to the data of p-cofilin-1, ABP gelsolin suffered a significant decrease in total protein expression levels for every sample group (Figure 5D). A tendency between these two showed the lowest fold decrease of 0.3 corresponding to PSP treatment before infection. Gelsolin data showed to be directing its focus in an opposite direction to results shown in Figure 4H.

Lastly, we examined the impact of C16-mediated inhibition on the IFN-IP PKR. As expected, phosphorylation of PKR significantly decreases with the implementation of C16 for every situation present (Figure 5E). This downward trend was observed to be directly opposite to PSP-treated cells with no inhibitory conditions from Figure 4E. However, a significant increase in total PKR was seen for every group present with the highest fold difference of 8.77 for PSP treatment before viral infection (Figure 5F). The increments established in total PKR data account for a minimum of 2-folds for all groups with an exception for HIV/PSP which amounts to an increase of ±1.4-fold compared to our data from Figure 4D. These results may imply an accumulation of total protein in the cell. In summary, inhibition of PKR activation significantly decreases cofilin-1 phosphorylation as well as gelsolin protein expression in an inversely proportional manner. Consequently, the reduction of PKR phosphorylation leads to an increase in the total protein counterparts of PKR and cofilin-1.

### 3.6. Inhibition of Endoribonuclease Activity of IRE1α Is Associated with De-Regulation for Both PKR and Cofilin-1 Phosphorylation

**Figure 6:**
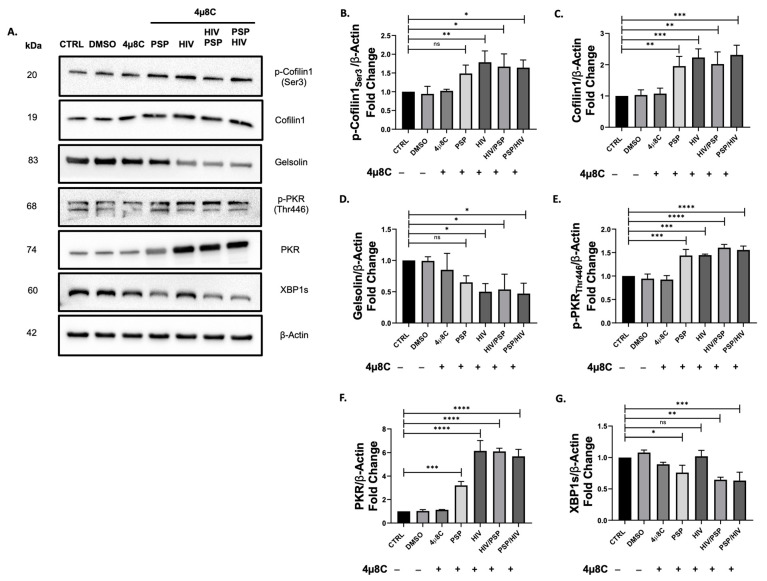
IRE1α modulates downstream phosphorylation activity of PKR and cofilin-1. Endoribonuclease activity of IRE1α was suppressed after 221.8 nM of 4µ8C inhibitor in THP1 monocytic cells with or without PSP treatment. (**A**) Representative Western blot data for the protein expression levels of cytoskeletal: (**B**) pCofilin1; (**C**) Cofilin1; (**D**) Gelsolin; IFN-IP: (**E**) p-PKR; (**F**) PKR and UPR: (**G**) XBP1s. β-Actin was used as a loading control and for normalization of data. Images were quantified using the ImageJ software (NIH, version 1.52a) by comparing the integrated density value with the control group. Mean ± SEM and significant difference (*), *p* ≤ 0.05, (**), *p* ≤ 0.01, (***), *p* ≤ 0.001, (****), *p* ≤ 0.0001 are shown and were determined using one-way ANOVA with Tukey multiple comparisons test, *n* = 3. All Western blot images can be found in Figure S2.

Previous studies have shown that the induction of UPR has had a history of cross-talking with IFNs, specifically PKR [61,63,70]. Additionally, our viral load data have revealed an increase in HIV-1 entry when IRE1α endoribonuclease activity is blocked (Figure 3). Since PKR inhibition led to a significant decrease in p-cofilin-1 and inverse results in its total counterparts (Figure 5), we aim to unveil the potential role of IRE1α in these signaling components. Cells were treated with 221.8 nM of the endoribonuclease blocker 4µ8C for a period of 5 h prior to infection and treatment as previously performed in our methodological approaches. Immunoblot assays have revealed a significant increase in the phosphorylation activity of cofilin-1 in every experimental group (Figure 6B). This regulatory behavior resulted in higher fold expression values than C16 data (Figure 5B) but remained lower in comparison to PSP-treated cells with no drug intervention (Figure 4G). THP1 cells before infection demonstrated a ±50% average fold-change reduction in the midst of 4µ8C compared to no drug treatment. Interestingly, total cofilin-1 protein levels also received an increase in fold change. PSP-treated cells before viral infection obtained the highest fold value of 2.31 on average compared to the control group (Figure 6C). According to what we reported in Figure 4F and Figure 5C, 4µ8C triggered elevated levels of protein expression compared to where no drug was administered but remain lower when cells were exposed to C16. Gelsolin levels were analogous to our results in Figure 5D, favoring a significant downward trend for all groups (Figure 6D). The lowest fold value of 0.47 on average was seen for PSP/HIV samples.

In relation to IFN-IP, we sought to investigate the molecular mechanism behind the regulatory effects of IRE1α and PKR. Our data showed that phosphorylation of PKR had a similar impact on the protein levels of phosphorylated cofilin-1. The expression values of p-PKR endure the inhibitory effects of 4µ8C and in this regard, it remained higher than C16 (Figure 6E). Contrary to what was observed in our data from Figure 4E, this same regulation was inferior where no drug activity happens. The total counterpart PKR would maintain elevated levels (Figure 6F) in contrast to no inhibitor (Figure 4D). However, the same regulatory position did not meet the same standards as its C16 equivalent (Figure 5F), by experiencing overall lesser protein levels across all experimental groups. When compared to the total cytoskeletal marker cofilin-1, both markers’ levels increased during 4μ8C inhibition.

To corroborate that the observed results were directly involved with the activity of 4µ8C as well as to validate that our chosen concentration inhibited IRE1α endoribonuclease activity, the UPR marker X-box-binding protein 1 spliced (XBP1s) was included in our Western blot approach. XBP1 is a direct splicing effector downstream of the IRE1α endoribonuclease signaling cascade [71]. A reduction in XBP1s was observed for all experimental situations relative to control, insinuating a 4µ8C-mediated inhibition towards the specific RNase activity of IRE1α (Figure 6G). Taken together, this data is indicating a shift in protein levels and active roles of PKR and cofilin-1. Most importantly, the inhibition of IRE1α is showing a linkage and a closely related pattern between these two markers.

### 3.7. PSP Regulates the Gene Expressions Associated with Cytoskeleton and IFN-IP Signaling

**Figure 7:**
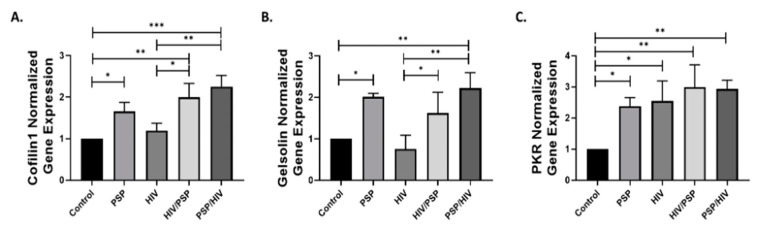
Validation of western blot results using RT-qPCR analysis for cytoskeletal and IFN-IP markers. Relative gene expression analyzed through RT-qPCR approach. The mRNA expression levels for the ADF: (**A**) Cofilin-1; ABP: (**B**) Gelsolin; and IFN-IP: (**C**) PKR are shown. All data were normalized using 18S as a housekeeping gene in response to PSP treatment. Mean ± SEM and significant difference (*), *p* ≤ 0.05, (**), *p* ≤ 0.01, (***), *p* ≤ 0.001 are shown and were determined using one-way ANOVA, with Tukey multiple comparisons test, *n* = 3.

Since both phosphorylated/total protein expressions of PKR and cofilin-1 are highly overexpressed in PSP-treated cells, we investigated the function of PSP as a regulator of the genes associated with these two signaling pathways. We hypothesized that the active shifting of total and phosphorylated forms of these markers, observed in preceding data, is partially due to the influence of PSP at the gene level. In addition, we decided to incorporate gelsolin in our dataset due to its seemingly tied role in protein regulation with PKR. We performed RT-qPCR analysis for PSP-treated cells under all conditions established in previous experiments. Supplementation of PSP increased the gene expression of cofilin-1 in all treated sample groups with PSP/HIV having the highest fold average of 2.25 (Figure 7A). A slight increase was observed for the infection group alone; however, it proved to have a non-statistical significance compared to the control. Gelsolin has been shown to have a similar gene regulative pattern as cofilin-1, where PSP addition incremented the overall levels of this protein (Figure 7B). The gene expression analysis identified significant upregulation of PKR in all groups present (Figure 7C). Concurrent with our Western blot experiments, RT-qPCR has revealed a close regulatory pattern for all signaling markers under PSP presence. Overall, this data opened a new insightful interpretation for PSP-induced signaling markers. We summarize our findings in Figure 7A–C, as PSP demonstrates control over PKR and gelsolin at the gene level and most importantly, the key HIV-1 entry factor, cofilin-1.

## 4. Discussion

Natural products have been wildly used in research as remedies to counteract the effects of diseases, particularly HIV-1. Compounds such as tea polyphenols show great potential at directly inhibiting HIV-associated malignancies [72]. Other organic substances, namely high-mannose oligosaccharides amalgamate, have demonstrated potent antiviral activity by blocking HIV-mediated entry [72,73]. Additional studies have elucidated the roles of carbohydrate-binding agents originating from plant lectins that significantly impact viral infection [74]. Having said this, PSP is a natural and commercially accessible supplement from the mushroom *Coriolus versicolor*. Previously, we published novel findings elucidating the anti-HIV effects of PSP in in vitro HIV-infected THP1 monocytic cells and ex vivo PBMCs. PSP has been demonstrated to possess anti-replicative capabilities against HIV-1 by promoting the upregulation of antiviral chemokines such as RANTES, MIP-1α/β, and SDF-1α. [8]. These chemokines are known to inhibit viral replication and as a result, they serve as immune enhancers [75,76,77]. Our laboratory has pioneered the immunomodulatory properties of PSP through a TLR4 response, giving way to unexplored signaling pathways that may further explain its immune-boosting factors. Moreover, viral load results have given insightful meaning to the internal antiviral effects that PSP possesses. The average inhibition percentage reported in acutely infected THP1 cells was 61%. This publication served as the basis for proposing this current study since the external role of PSP on HIV-1 entry currently remains unidentified. In addition to its immunological effects, PSP has been incorporated as an anti-cancer medication in clinical approaches with proven efficiency [78]. Due to its natural characteristics, no adverse side effects have been established with the use of PSP. Our research groups have previously performed cytotoxic assays which resulted in no significant signs of toxicity at concentrations higher than the established experimental dose. This essentially confirms the literature research data. Studies have shown that PSP has the innate ability to significantly increase the total count of immune cells without the unfavorable side effects seen in HAART [78,79]. Concurrent with the given history of PSP, other researchers have shown its potentiality with gastral and esophageal cancers due to its characteristics of showing a superior CD4+ and CD8+ cell count among other treatments [80].

The HIV-1 co-receptors CXCR4 and CCR5 signal directly through the cytoskeleton to promote viral entry into host-immune cells (Figure 1). It has been well-stated in the literature that HIV-1 requires manipulation of the actin filaments to promote receptor clustering of CXCR4/CCR5 and increase their spatial orientation and interactions as a pre-entry requisite. However, these receptors alone are not sufficient for infection to take place and pose a challenge for efficient fusion and insertion of the viral capsid. This is mainly due to actin filaments functioning as a physical barrier and blocking HIV-1 entry [16,26,27,28]. A second factor that comes into play is membrane fusion between the target cell and the virus acting as a means for successful infection in a post-entry step manner [16]. The regulator of these processes can be attributed to cofilin-1 by shifting the actin dynamic complex through the phases of elongation and breakdown. Specifically, this signaling cascade is modulated by the inactivity of phosphorylated cofilin-1 and its activated form respectively [81,82]. It is for these reasons that HIV-1 must overcome this restrictive property for successful infection to occur. Studies have shown that this HIV-induced mechanistic approach has been postulated as a delicate process. Therefore, interference with either form of cofilin-1 leads towards viral restriction [21,22]. In addition, gelsolin has also been associated as a deciding factor for HIV-1 entry, given that it shares the same regulation over cytoskeleton dynamics. Due to this, it is a target of interest in HIV-1 studies.

Given the knowledge of HIV-1 infection, this study investigated the role of PSP in regulating the ADF cofilin-1 required for viral entry through the IFN-IP and UPR signaling. We revealed numerous novel aspects of the external function of PSP as a regulator of viral entry. Among them, we showed that PSP has the potential to hinder HIV-1 entry by an approximately 74% when subjected to its antiviral effects before infection occurs in our in vitro model. The current research also highlights the underlying mechanisms by which PSP induces a shift in the phosphorylation states of PKR and consequently cofilin-1. It has been postulated in the literature research that both PKR and IRE1α can overlap with each other [50]. Inhibitory experiments in our dataset and validated with two pharmaceutical blockers in THP1 cells have confirmed these studies. The present research has demonstrated that PKR and IRE1α seem to have an established correlation under PSP influence. At the same time, inhibition of these biomarkers has led to a significant increase in viral entry for both cases. This suggests that the restrictive nature of PSP is associated with these two molecular signatures.

In this research, we have applied quantitative proteomics analysis to understand the mechanistic pathways that are being influenced by PSP. The current data shows that PSP induces overwhelming deregulation of 111 cytoskeletal as well as 28 proteins pertaining to the UPR. Among these, crucial proteins such as PKR and IRE1α demonstrate favorable up-regulatory roles. Other critical components relating to the small GTPase family specifically required for HIV entry are seen in unfavorable regulatory positions. The indicated GEFS includes RAB3IP, RAB8B, RALGPS2, RASAL2, RASGRP2, ARHGAP 15 and 17. The functions of the referred GEFS are known to signal the exchange of GDP to active GTP, upstream of cofilin-1 activity, and result in cytoskeleton remodeling [83,84,85]. With significant weight, we highlight the downregulation of SSH3 phosphatase, to which HIV signals through its co-receptor to promote the second phase for capsid fusion [18,19]. This strongly suggests that PSP is signaling toward the regulation of cofilin-1. Additional evidence suggests that PSP exerts control over actin dynamics through the upregulation of tropomyosin and tropomodulin, which directly binds and regulates actin-filaments lengthening [57,58,59]. Multiple studies have shown that UPR and cytoskeleton signaling overlap with each other and some of these characteristics can be attributed to the upregulation of PDI [40,41,42,43]. Its overexpression is mainly associated with disulfide bond formation and protein folding. However, this ER marker is also linked to the direct binding of β-Actin to regulate cytoskeleton reorganization [86]. Taken together, our proteomics data firmly implies that PSP has influence over actin dynamics and UPR signaling. Interestingly, a study has shown the relationship between the cytoskeleton and IFN-IP, particularly with PKR. The referred research demonstrated that PKR could serve as an upstream regulator of cofilin-1. Specifically, PKR can phosphorylate cofilin-1 and render it inactive [54]. Due to our gathered results and documented facts, we were interested in investigating the specific regulatory role of cofilin-1 as well as its correlation with PKR. In particular, PSP-treated cells resulted in an overexpression for both total and phosphorylated forms of cofilin-1 as well as for PKR. Given the literature knowledge that we possess between these two molecules, we proposed that cofilin-1 regulation is being influenced in some way by PKR and essentially can serve as a primary restrictive pathway. To challenge this hypothesis, we directly inhibited the ability for PKR to be activated by either auto-phosphorylation or indirectly stimulated by other factors such as foreign double-stranded RNAs and the endoribonuclease activity of IRE1α. Interestingly, PKR inhibition has led to a significant reduction of phosphorylation activity of cofilin-1 comparable to the control. On the opposite side of the spectrum, there was an increase in total cofilin-1 with a superior expression than PSP-treated cells with no drug intervention. This data suggests that PKR has a strong role in dictating the active state of ADF/cofilin-1 under the influence of PSP.

The ABP gelsolin is known for its abundance and functions in actin capping and severing. To date, this cytoskeletal marker shares three isoforms known as plasma (Isoform 1), cytoplasmic (Isoform 2), and gelsolin-3 [87]. A particular study from Irving et al. has published that PKR is able to counteract viral entry in the innate immune system by directly binding and inhibiting gelsolin [88]. This study raised interest in the interactions between this cytoskeletal marker and PKR since our data reveals both overexpression and restrictive properties of this IFN-IP. Our quantitative proteomic analysis has given insight into the downregulation of gelsolin-3. Since cytoplasmic gelsolin is a primary interest in HIV-entry studies due to its involvement with cytoskeletal events and its role has not been elucidated in PSP before, we decided to incorporate this marker in our experiments to elucidate its role in PSP-induced cells. In contrast to gelsolin-3, PSP seems to have some up-regulatory properties when it comes to its counterpart, showing elevated levels for every experimental condition. When PKR activity was blocked, gelsolin also showed analogous expressions in fold change when compared to p-cofilin-1, suggesting that the functional role of PKR is overlapping with cytoskeletal components. This dataset also serves as an additional validation marker where our chosen concentration for the C16 drug was effective at inhibiting PKR, given that both gelsolin and p-PKR were affected. When taken together, these data heavily suggest that PSP-induced overexpression of PKR is extending its reach by regulating pivotal viral entry components.

It is well known that HIV is recognized by the IFN response and triggers a high production of type I IFNs [89]. This is also observed when THP1 cells were infected, which resulted in a 3.4-fold increase. PKR is expressed at relatively low levels under normal conditions and its transcription begins once type I IFNs are stimulated by viral replication [90]. This study shows that both PSP and HIV-1 independently trigger a high expression of PKR with a greater effect seen when both conditions are combined. When cells were treated with PSP before infection took place, a 4.31-fold average increment of PKR was seen. This group constitutes the same that partook in our viral load analysis. Therefore, since PKR has been postulated before as a restrictive factor for viral entry, our validated results further strengthen these views indicating that PKR plays a major role in hindering viral entry in THP1 cells.

In recent years, studies on the interactions between UPR and PKR have surged with the purpose of promoting an anti-pathogenic environment. Among them, NF-κB signaling and production of type I IFNs have been researched to gain insights into their respective roles such as survivability and immune response [49,91,92]. Interestingly, a study has also shown that IRE1α directly activates PKR in response to Chlamydia trachomatis infection in a TLR4-dependent manner [50]. Having said this, our previously published data stand out by demonstrating the anti-viral effects of PSP through TLR4 and NF-κB. The present research has also demonstrated and validated UPR activity for the first time in PSP-treated cells. Specifically, we saw that PSP highly upregulates the expression of IRE1α by 2 folds while HIV-1 infection seems to minorly lower this response alone or in combination. Given the history of IRE1α in relation to the immune system and PKR, we aim to unveil its specific role in both viral entry and its association with cofilin-1. The gathered data indicates that IRE1α can regulate, to an extent, the phosphorylation patterns of both PKR and cofilin-1. This modulation remains significantly higher than the data seen in the C16-PKR blocker. However, inhibition of IRE1α also led to a decrease in the activity of p-PKR/p-cofilin-1 compared to PSP-treated cells with no drug activity. This renders the effects of this blocker in the middle ground between C16 and PSP non-inhibition, suggesting that PSP-induced IRE1α can serve as an upstream regulator. A peculiar pattern was observed where blockage of IRE1α endoribonuclease activity resulted in elevated levels of PKR/cofilin-1 total counterparts similar to C16. Consequently, gelsolin was also affected by a significant decrease in overall levels comparable to C16 data, suggesting that the overexpression of PKR is once again affecting the regulation of gelsolin. Since we have a clearer insight into the roles of IRE1α in PSP treatment, we were interested if PSP is inducing acute or chronic UPR/ER stress through GRP78. PSP has shown to have no statistical difference in GRP78 compared to the control group. On the other hand, HIV-1 infection significantly increases its overall expression across all groups. In hindsight, while HIV-1 shows superior GRP78 production, an established pattern was observed where PSP actively lowers these levels in all instances where it is present. This indicates that PSP is favoring acute rather than chronic ER stress, by counteracting the apoptotic effects of HIV-1 through GRP78 downregulation. This is evident by the effects of PSP addition before HIV infection which resulted in a 0.81-fold difference compared to 1.83 in the HIV-1 group alone. Additionally, these data are showing concurrency with previous studies as well as our published data, which emphasizes the non-toxic effects of PSP.

**Figure 8:**
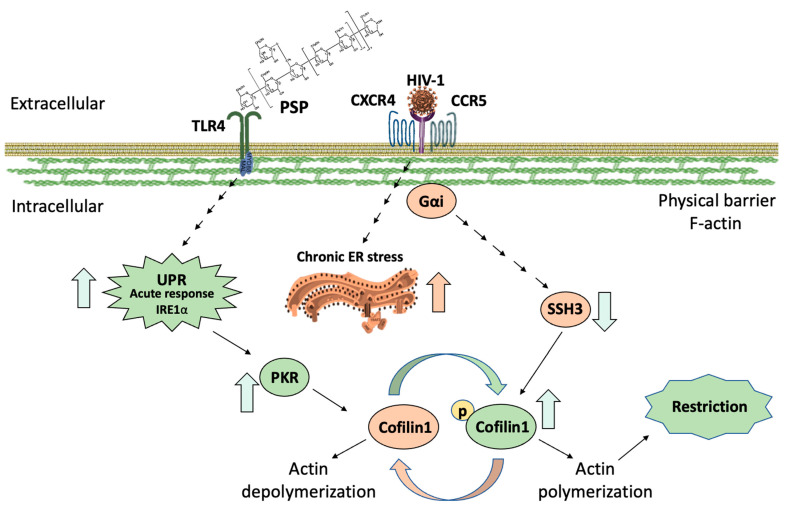
Model depicting PSP signaling through a UPR/IFN-induced pathway. HIV-1 infection results in a chronic ER stress response, while simultaneously dephosphorylating cofilin-1 through SSH3 phosphatase. This results in actin depolymerization for viral entry. Prior to infection, PSP treatment induces actin polymerization via an acute UPR. PKR mediates downstream phosphorylation of IRE1α signals and reverses HIV-induced actin remodeling while infection persists. Legend colors: Green—PSP-mediated signaling and events. Cream- HIV-downstream pathways.

We also evaluated the effects of PSP at the gene level due to our hypothesis that accumulation of total PKR and cofilin-1 was due to genetic disturbances. As we expected, PSP induces the upregulation of cofilin-1, gelsolin, and PKR during RT-qPCR analysis. This gives weight to our hypothesis that the overwhelming upregulation of these markers was due to gene influences during inhibitory experiments. In addition, this further explains the reason for both total and phosphorylated forms of PKR/cofilin-1 being in an up-regulatory position. Overall, the corresponding results have led us to propose a mechanistic model that highlights the role of PSP in lowering viral entry through IRE1α and PKR (Figure 8). The overlapping signaling between IRE1α and PKR would allow the immune system to implement the necessary conditions to block viral entry. Consequently, this would restrict HIV-1 at the early entry step due to the overwhelming inactivity of cofilin-1. In general, the study at hand uncovers potential targetable PSP/UPR/PKR pathways that can be implemented in broader research with the aim of future therapeutic approaches.

### Future Directions

PSP continues to show anti-HIV capabilities by currently targeting both entry and replicative cycles. Subsequently, to understand its true therapeutic potential, a comprehensive study will be conducted on the adaptive immune response. Specifically, future research will be validated using in vitro/ex vivo models as well as a population consisting of healthy and HIV-infected subjects.

## 5. Conclusions

Our study demonstrated, for the first time, that PSP hinders HIV-1 entry before infection takes place. Furthermore, we have shown that this restrictive ability is associated with positive regulation of PKR activation as well as IRE1α endoribonuclease activity. Consequently, cofilin-1 phosphorylation is also affected by these two signaling markers and it is reflected in the early entry phase. The present study has provided novel mechanistic insights between the interplay of UPR, IFN-IP, and cytoskeletal events. Taken together, the data provided here suggest that PSP has the potential to be used as a natural alternative to target HIV-1 entry.

## Data Availability

The data supporting the results are found within the manuscript and Supplementary Files.

## Supplementary Materials

The following supporting information can be downloaded at: https://www.mdpi.com/article/10.3390/v15030804/s1, Figure S1: MTT cell viability of C16 and 4µ8C inhibitors. Figure S2: Full western blot images. Table S1: Differentially expressed cytoskeletal-related proteins identified in PSP treated THP1-Blue-CD14 cells. Table S2: Differentially expressed UPR proteins identified in PSP treated THP1-Blue-CD14 cells.
