# Supplementary material for: The Antiviral Compound PSP Inhibits HIV-1 Entry via PKR-Dependent Activation in Monocytic Cells
Source: Viruses. 2023 Mar 22;15(3):804. doi: 10.3390/v15030804 (PMC10051440; doi:10.3390/v15030804)
Supplement: Supplementary file 1 [file viruses-15-00804-s001.zip › viruses-2289446-supplementary.pdf]

A.

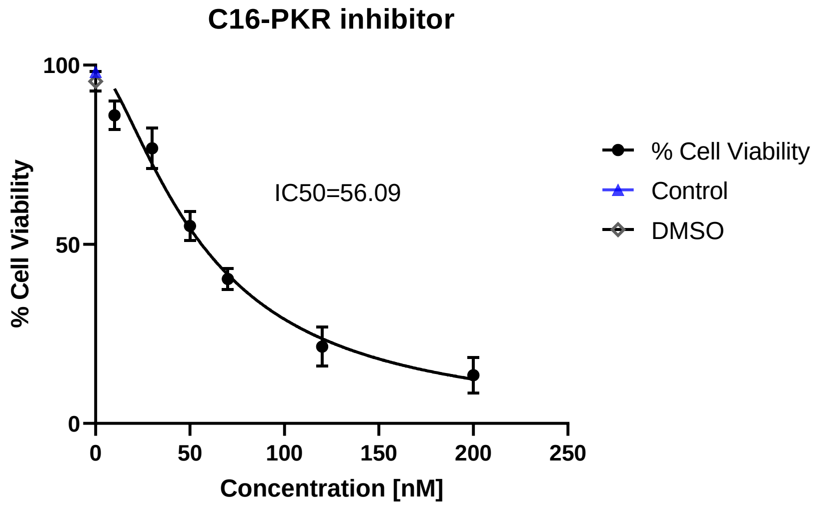

B.

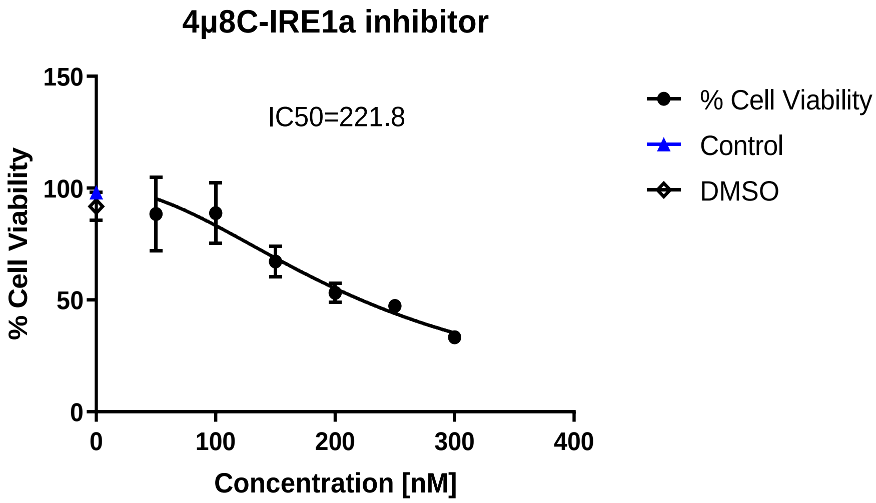

Figure S1. MTT assays for detection of IC50 of pharmacological drugs used. The concentration that induced 50% cell viability in THP1 cells were determined for (**A**). C16 (IC50 = 56.09nM) and (**B**) 4μ8C (IC50 = 221.8nM). Experiments were carried out in biological triplicates, *n*=3.

Figure S2: Full, original, uncropped, representative western blot images within manuscript

Western blot photos found in Figure 4:

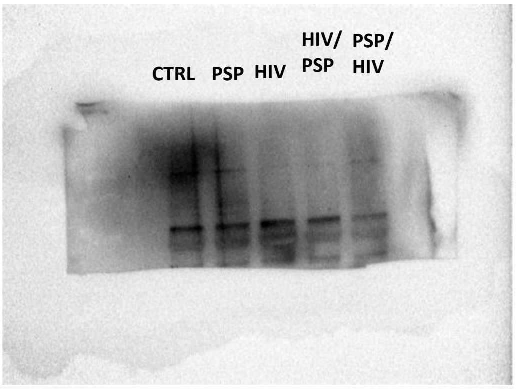

GRP78
78kDa

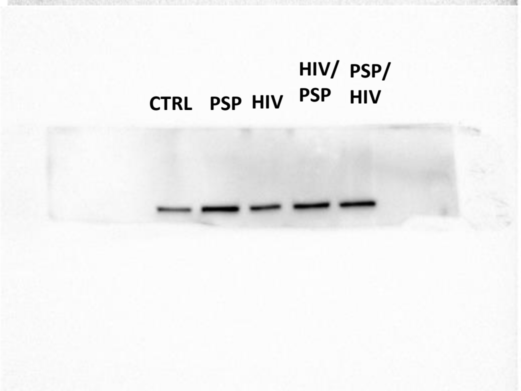

IRE1α
110kDa

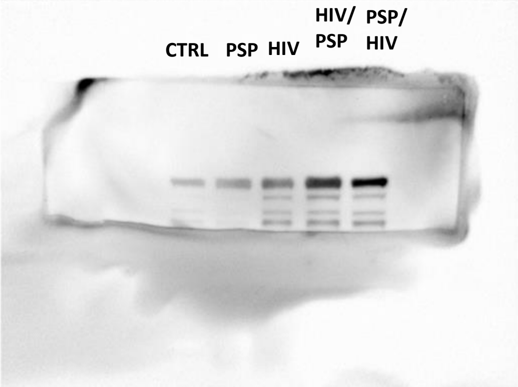

PKR
74kDa

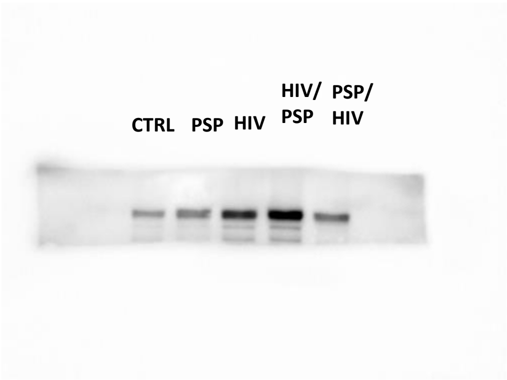

p-PKR (Thr 446)
68kDa

Original representative image of Cofilin1 found within manuscript.

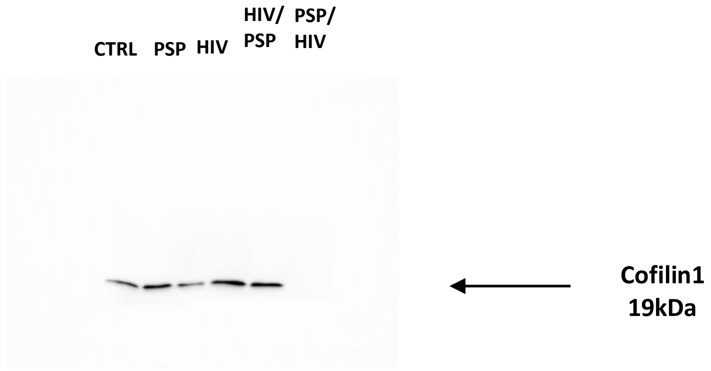

Same photo with contrast adjusted using Bio-Rad Image Lab software:

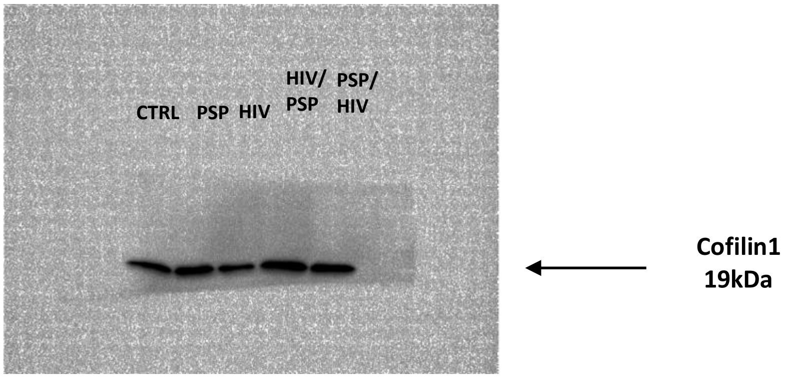

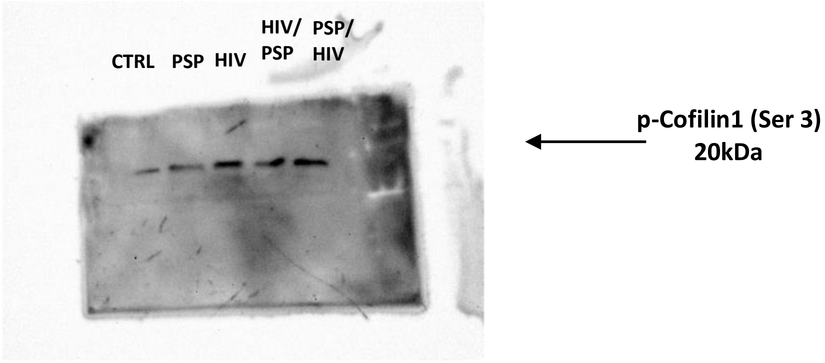

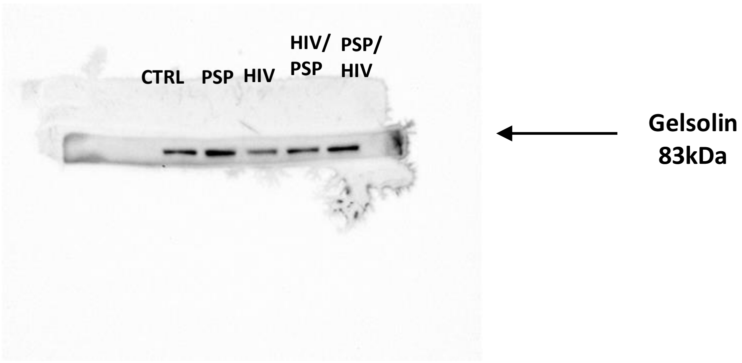

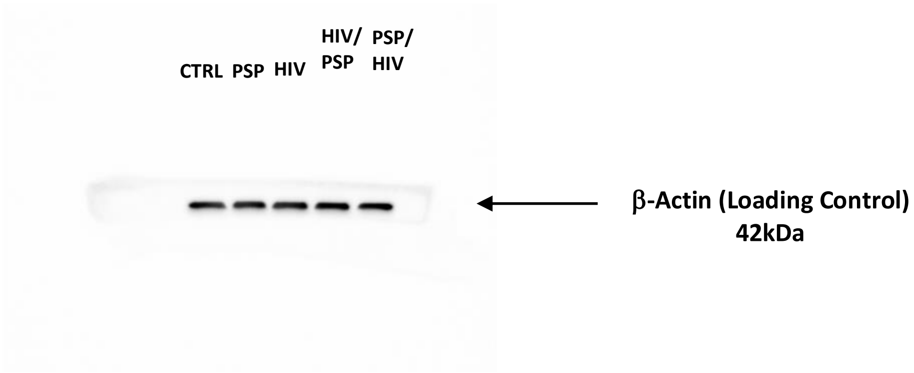

Western blot photos found in Figure 5:

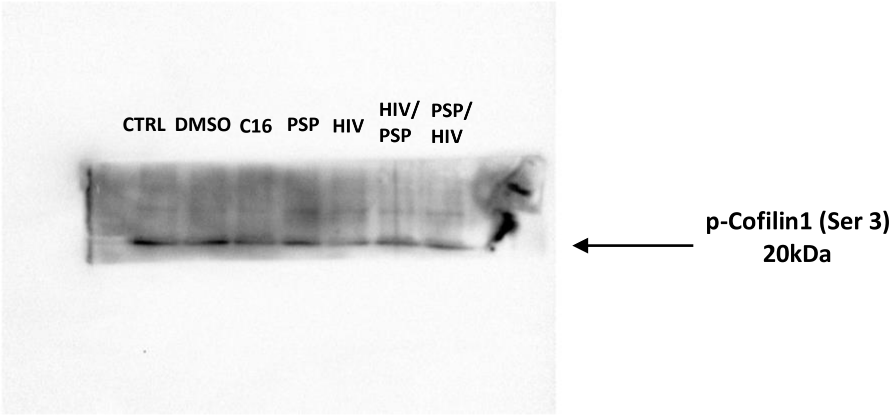

Original representative image for Cofilin1 found within manuscript:

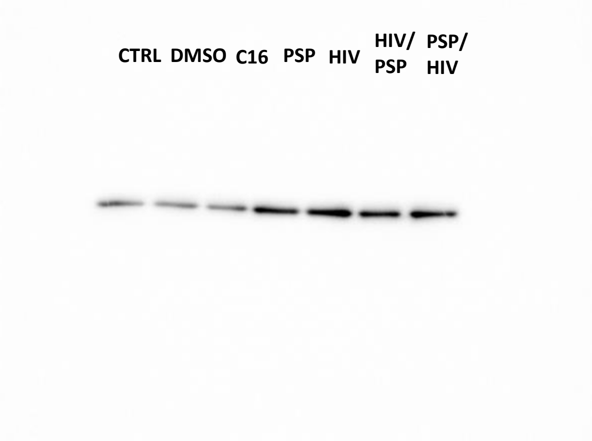

Cofilin1
19kDa

Same photo with contrast adjusted using Bio-Rad Image Lab software:

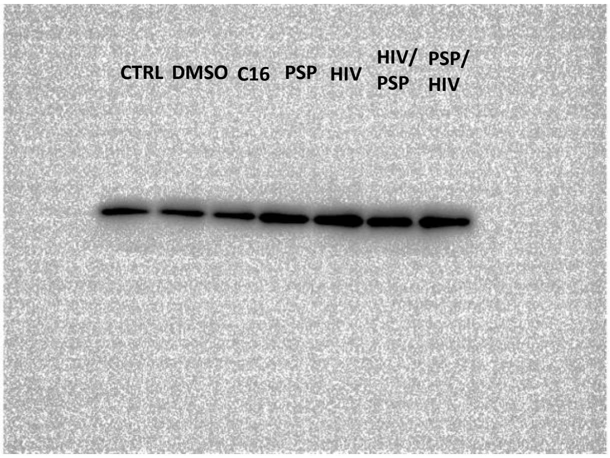

Cofilin1
19kDa

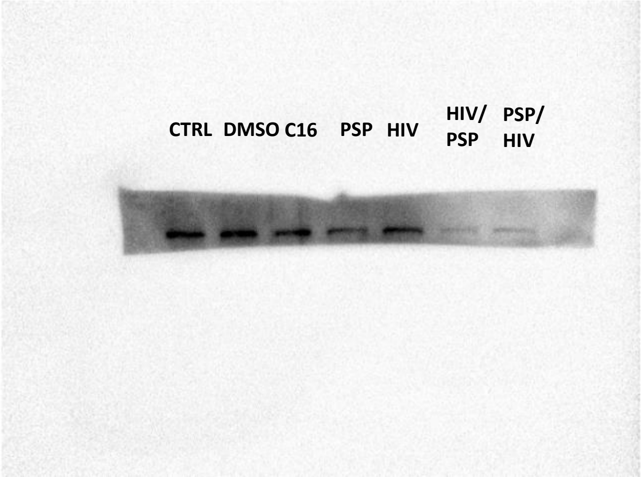

Gelsolin
83kDa

Original representative image of p-PKR found within manuscript:

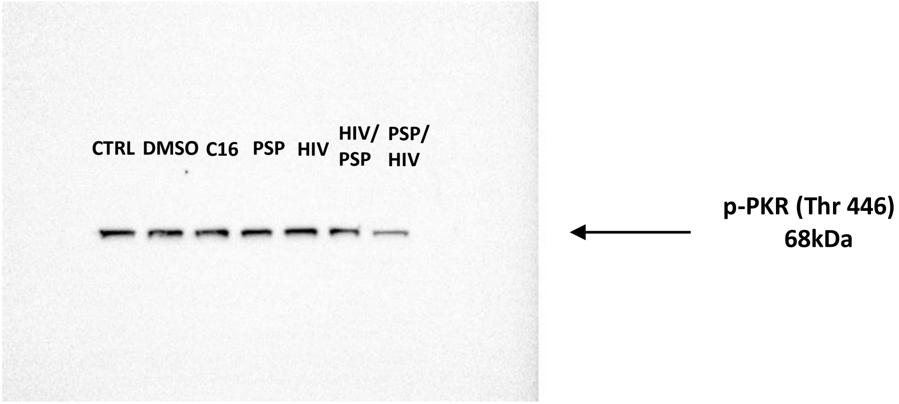

Same photo with contrast adjusted using Bio-Rad Image Lab software:

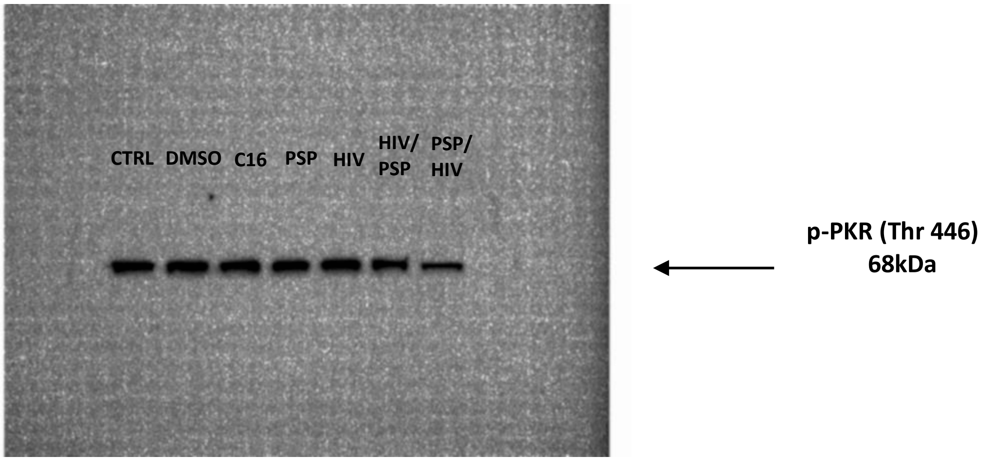

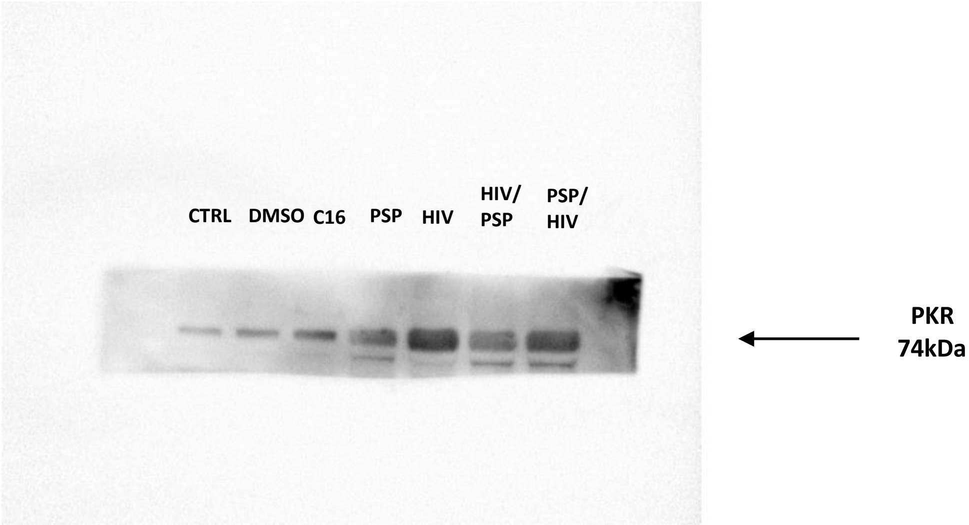

Original representative image of loading control found in manuscript:

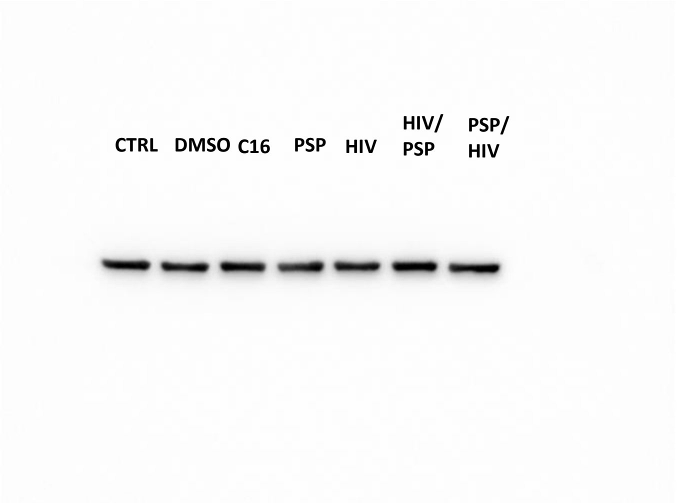

β-Actin (Loading Control)
42kDa

Same photo with contrast adjusted using Bio-Rad Image Lab software

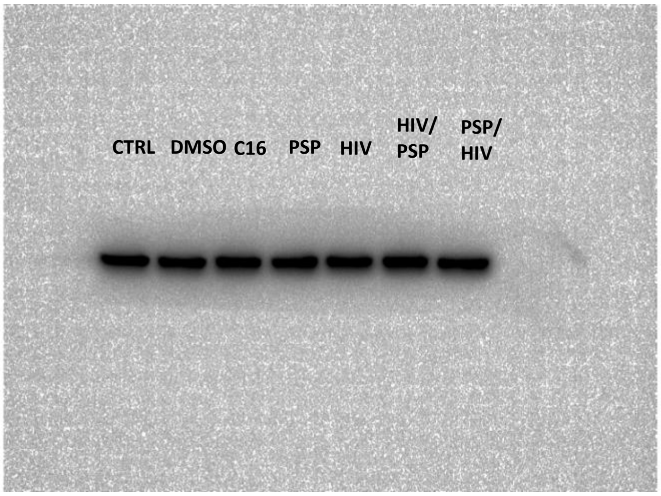

β-Actin (Loading Control)
42kDa

Western blot photos found in Figure 6:

Original representative image of p-Cofilin1 found in manuscript:

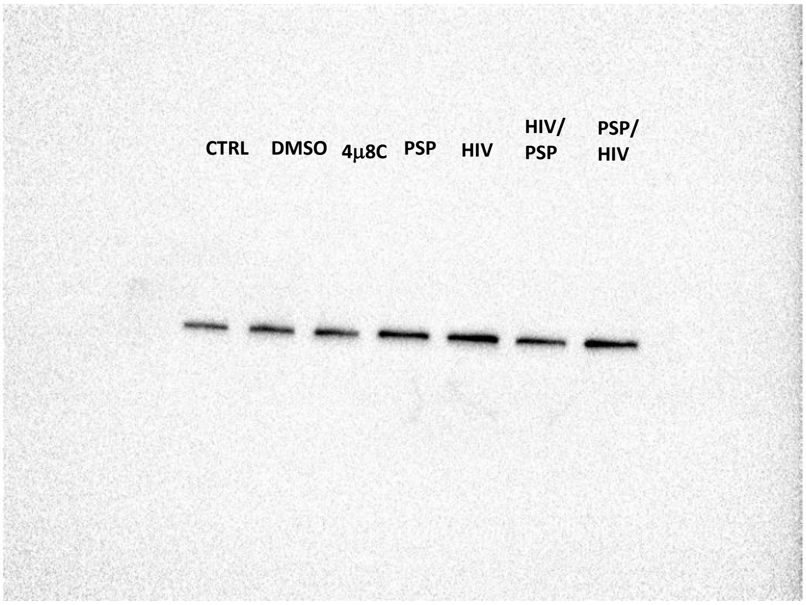

p-Cofilin1 (Ser 3)
20kDa

Same photo with contrast adjusted using Bio-Rad Image Lab software

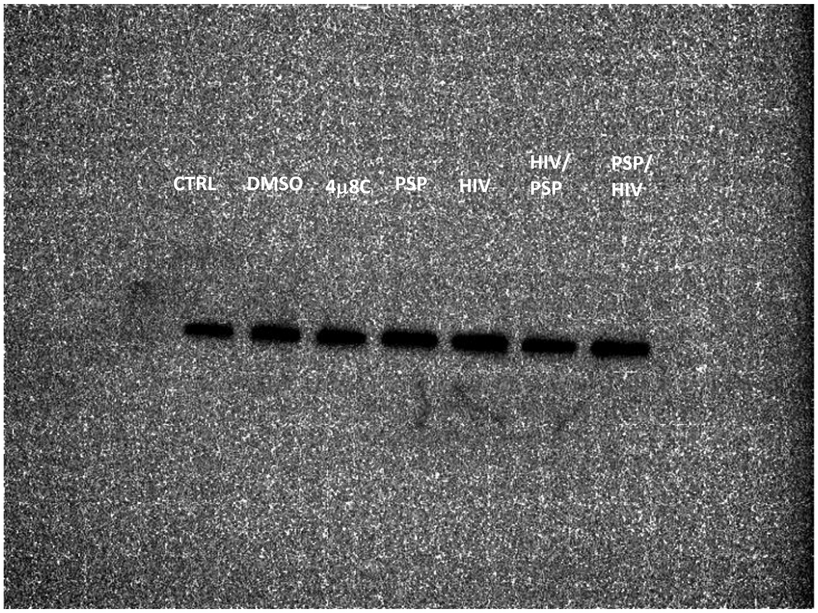

p-Cofilin1 (Ser 3)
20kDa

Original representative image of Cofilin1 found in manuscript:

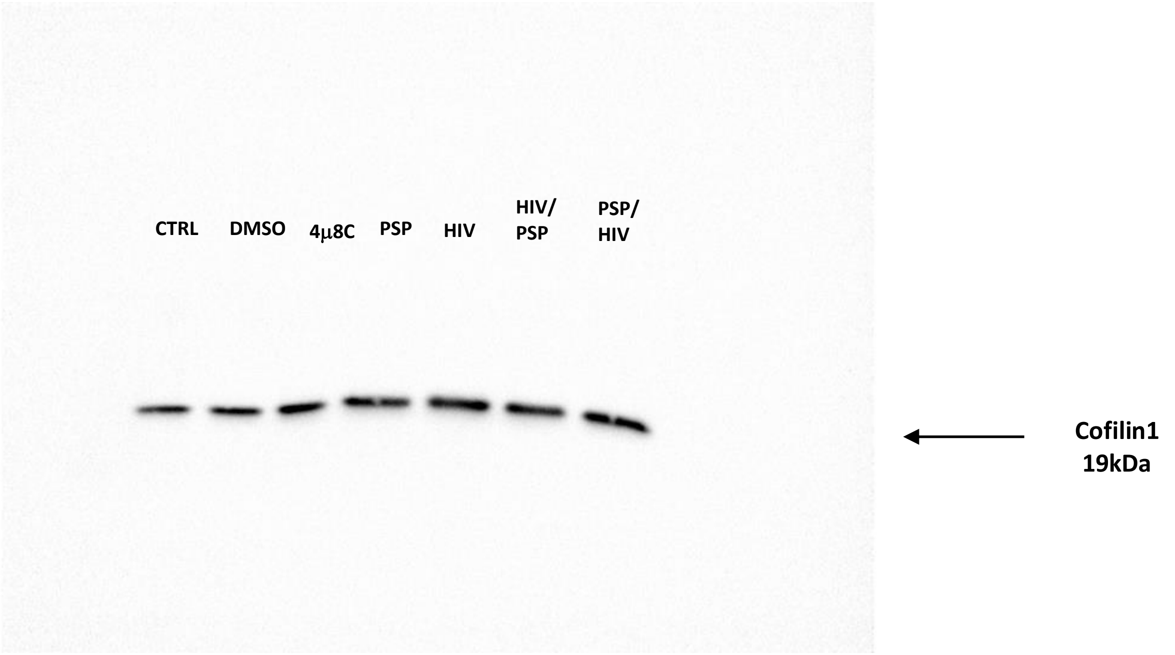

Same photo with contrast adjusted using Bio-Rad Image Lab software

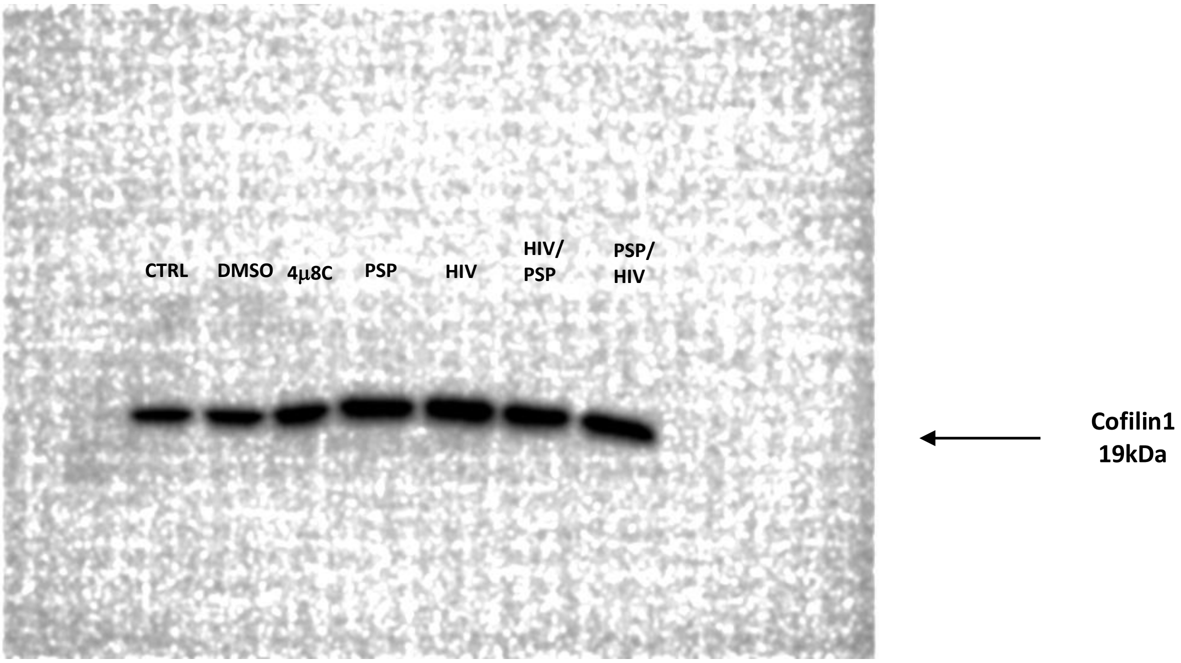

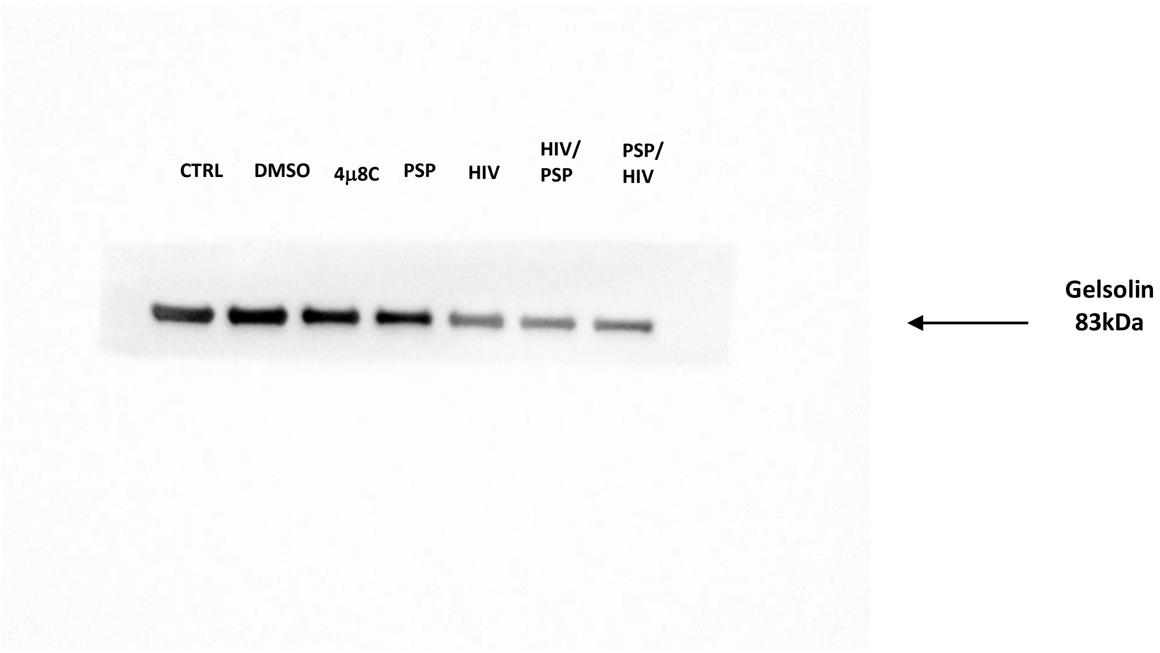

Original representative image of p-PKR found in manuscript:

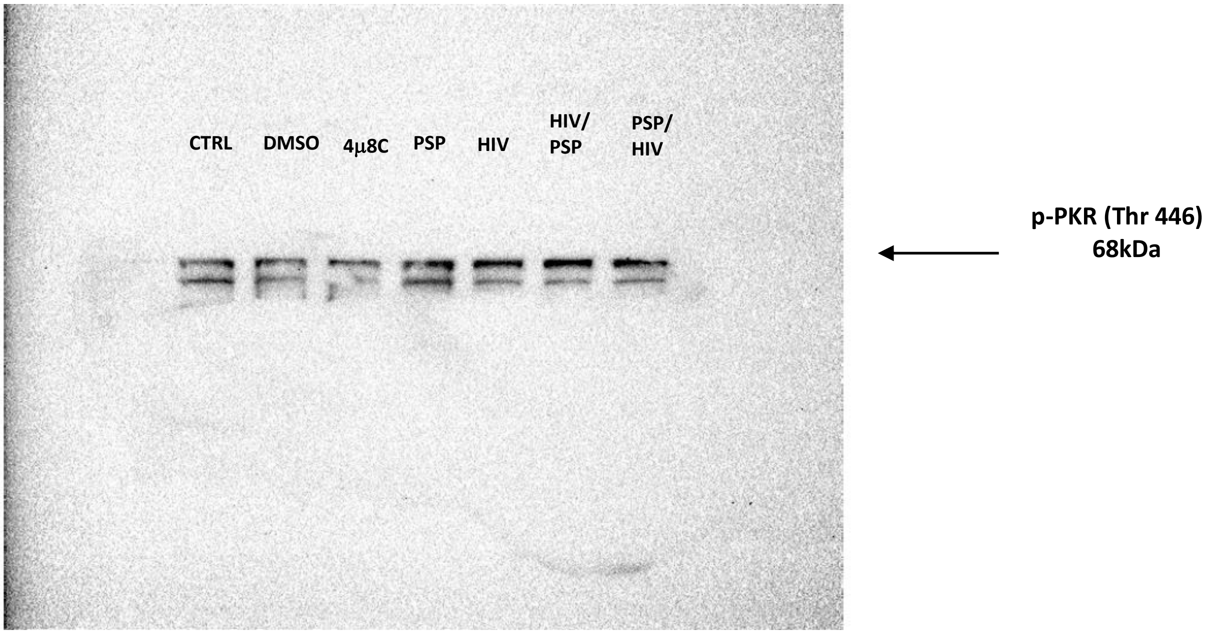

Same photo with contrast adjusted using Bio-Rad Image Lab software

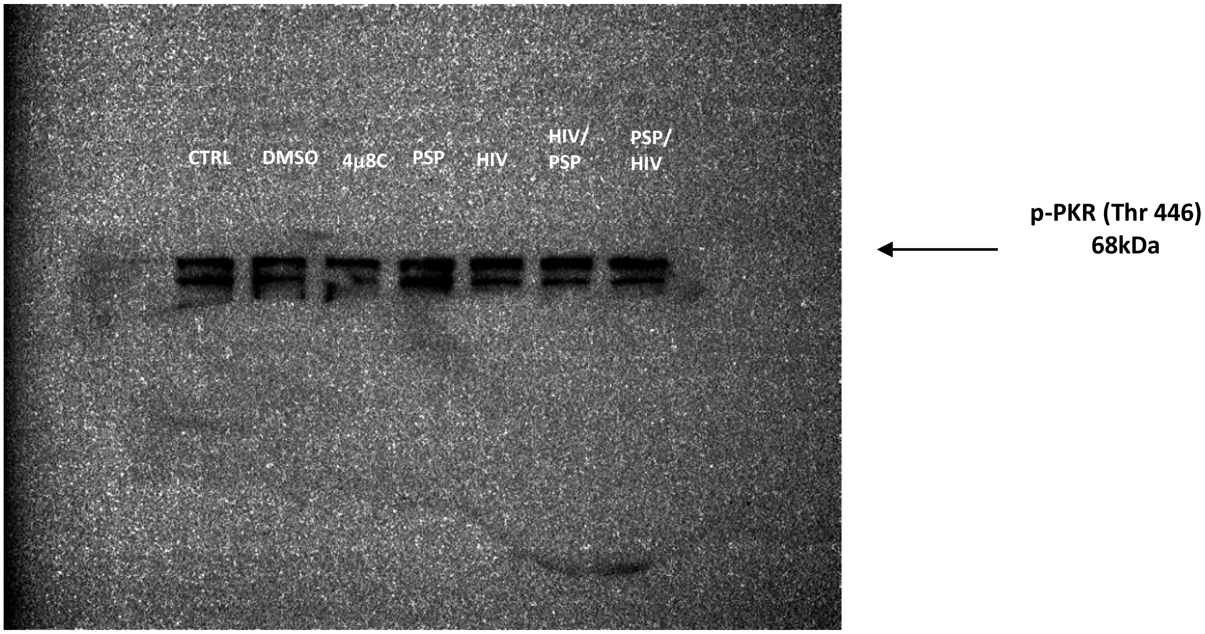

Original representative image of PKR found in manuscript:

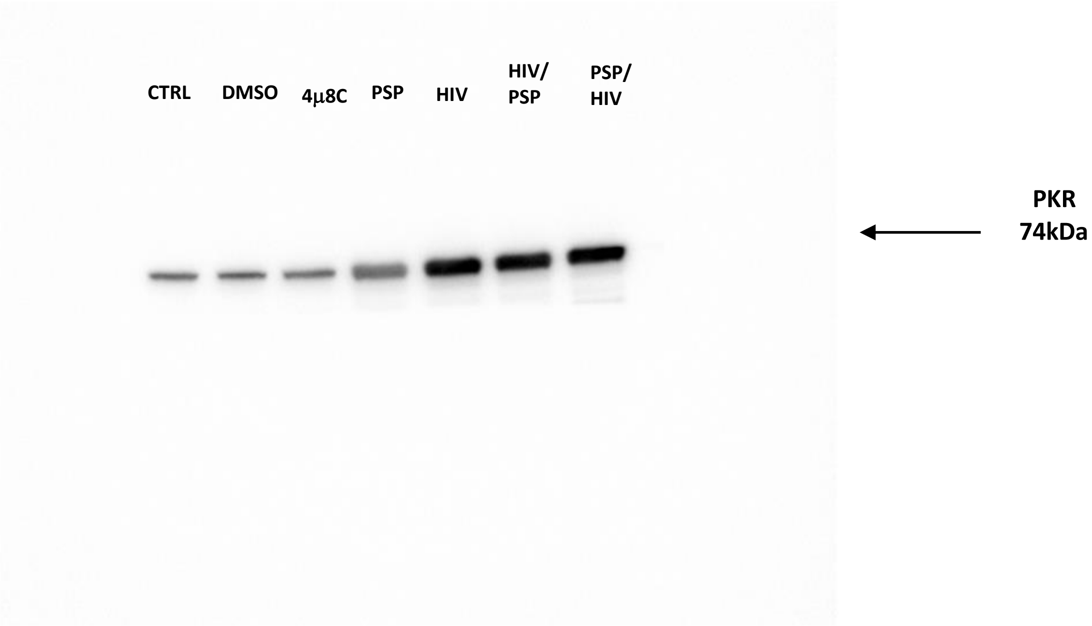

Same photo with contrast adjusted using Bio-Rad Image Lab software

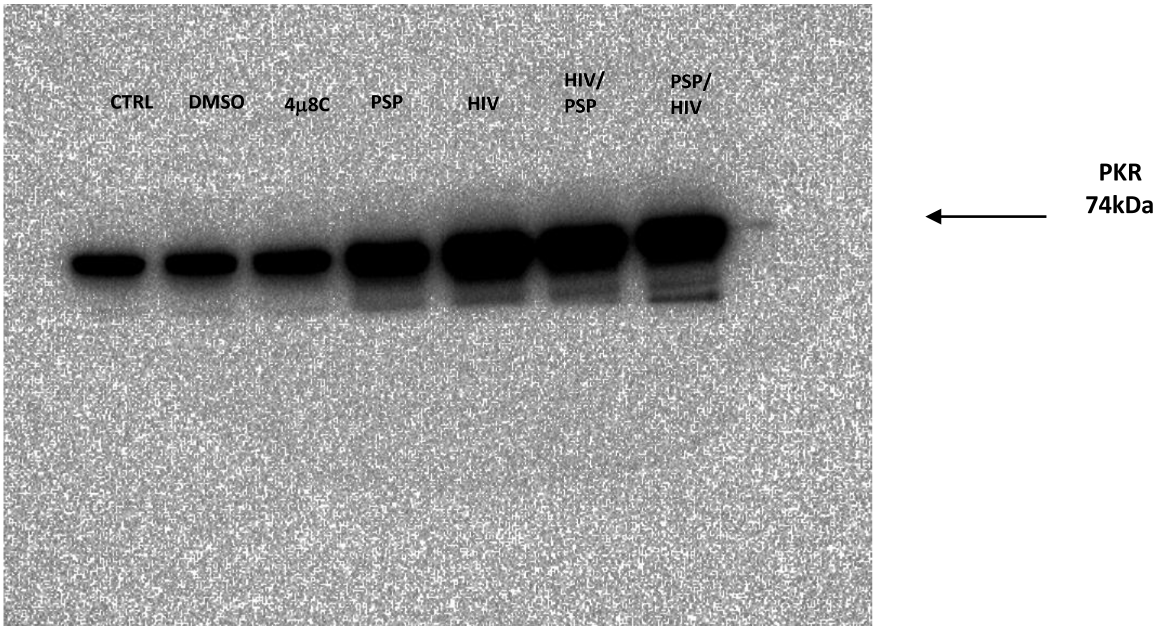

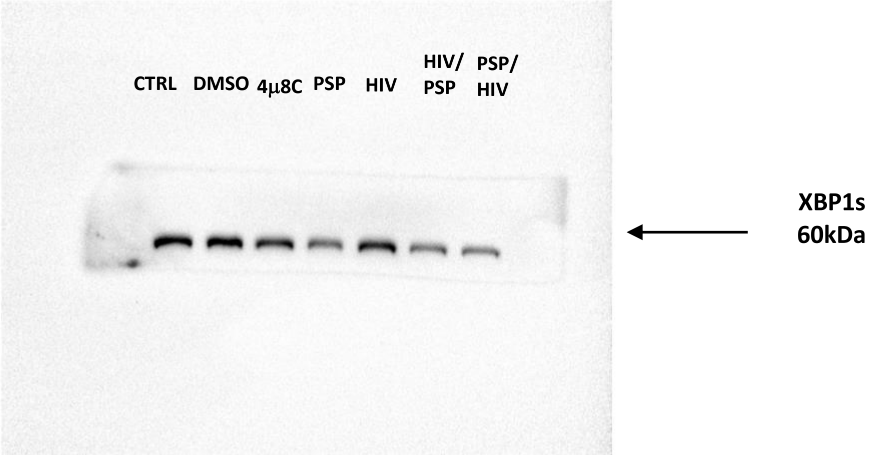

Original representative image of loading control found in manuscript:

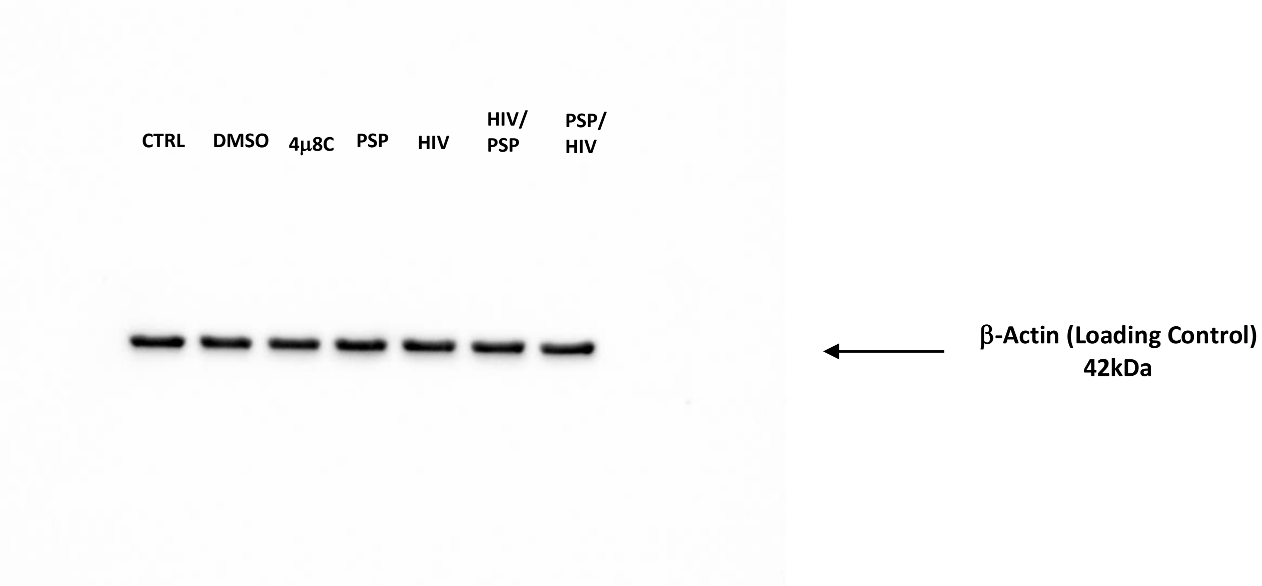

Same photo with contrast adjusted using Bio-Rad Image Lab software

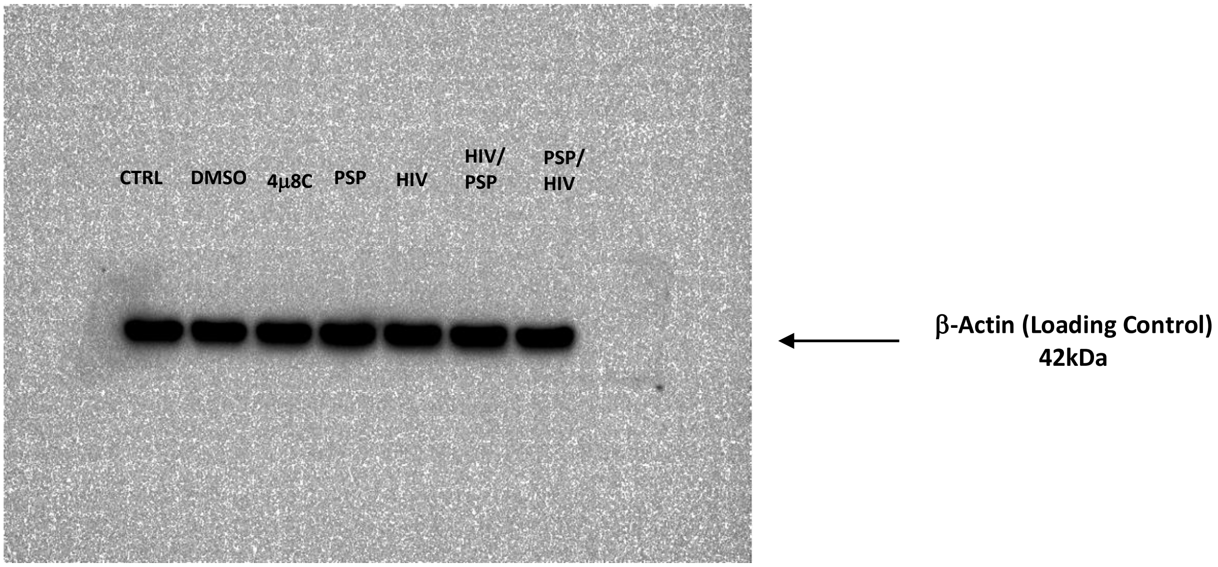

Table S1. Differentially expressed cytoskeletal-related proteins identified in PSP treated THP1-Blue-CD14 cells.

Accesion #	GENE	Quantified Spectra Counts	Protein Name	Average Expression in Control	Average Expression in PSP	Fold Change	Adjusted P Value
**Q9NRX4**	PHPT1	15	14 kDa phosphohistidine phosphatase	18.18	15.15	-1.20	0.0131
**P12814**	ACTN1	102	Alpha-actinin-1	18.19	15.15	-1.20	0.0005
**Q7Z5R6**	APBB1IP	25	Amyloid beta A4 precursor protein-binding family B member 1-interacting protein	18.91	14.42	-1.31	<0.0001
**P08133**	ANXA6	96	Annexin A6	14.40	18.94	1.32	<0.0001
**P02649**	APOE	5	Apolipoprotein E	13.64	19.70	1.44	0.0083
**P10415**	BCL2	5	Apoptosis regulator Bcl-2	20.07	13.26	-1.51	0.0462
**Q15121**	PEA15	4	Astrocytic phosphoprotein PEA-15	13.87	19.47	1.40	<0.0001
**P07384**	CAPN1	79	Calpain-1 catalytic subunit	18.31	15.02	-1.22	<0.0001
**Q99439**	CNN2	20	Calponin-2	18.51	14.83	-1.25	0.0134
**P48509**	CD151	3	CD151 antigen	15.12	18.21	1.20	0.0007
**Q9Y5K6**	CD2AP	37	CD2-associated protein	18.75	14.58	-1.29	0.0003
**P16070**	CD44	9	CD44 antigen	12.32	21.01	1.71	0.0001
**Q9NPY3**	CD93	10	Complement component C1q receptor	11.69	21.64	1.85	<0.0001
**O75956**	CDK2AP2	2	Cyclin-dependent kinase 2-associated protein 2	18.49	14.85	-1.25	0.0454
**Q5M775**	SPECC1	23	Cytospin-B	18.77	14.56	-1.29	0.0002
**Q16555**	DPYSL2	31	Dihydropyrimidinase-related protein 2	13.30	20.04	1.51	<0.0001
**Q9NQ86**	TRIM36	18	E3 ubiquitin-protein ligase	14.09	19.24	1.37	0.0049
**P98172**	EFNB1	2	Ephrin-B1	21.50	11.83	-1.82	<0.0001
**Q16658**	FSCN1	79	Fascin	14.46	18.87	1.31	<0.0001

**Q0JRZ9**	FCHO2	24	FCH domain only protein 2	18.62	14.71	-1.27	0.0037
**Q86UX7**	FERMT3	66	Fermitin family homolog 3	13.30	20.04	1.51	<0.0001
**O75955**	FLOT1	8	Flotillin-1	13.62	19.71	1.45	0.0024
**Q5T0N5**	FNBP1L	4	Formin-binding protein 1-like	19.56	13.77	-1.42	0.001
**O60234**	GMFG	15	Glia maturation factor gamma	18.56	14.78	-1.26	0.015
**P04792**	HSPB1	144	Heat shock protein beta-1	17.77	15.57	-1.14	0.0009
**Q9BYW2**	SETD2	18	Histone-lysine N-methyltransferase	18.76	14.58	-1.29	0.0018
**O00291**	HIP1	24	Huntingtin-interacting protein 1	9.99	23.34	2.34	<0.0001
**O75146**	HIP1R	4	Huntingtin-interacting protein 1-related protein	13.06	20.27	1.55	0.0265
**P05107**	ITGB2	82	Integrin beta-2	11.36	21.97	1.93	<0.0001
**P05362**	ICAM1	17	Intercellular adhesion molecule 1	1.41	31.93	22.68	<0.0001
**P19525**	EIF2AK2	29	Interferon-induced, double-stranded RNA-activated protein kinase	9.69	23.65	2.44	<0.0001
**Q9UI08**	EVL	26	Isoform 1 of Ena/VASP-like protein	18.48	14.86	-1.24	0.0005
**Q9UEY8**	ADD3	8	Isoform 1 of Gamma-adducin	20.31	13.02	-1.56	<0.0001
**P07355**	ANXA2	42	Isoform 2 of A	12.50	20.84	1.67	<0.0001
**Q8N556**	AFAP1	6	Isoform 2 of Actin filament-associated protein 1	13.33	20.00	1.50	0.0002
**Q8NCM8**	DYNC2H1	2	Isoform 2 of Cytoplasmic dynein 2 heavy chain 1	18.96	14.37	-1.32	0.007
**O95466**	FMNL1	47	Isoform 2 of Formin-like protein 1	14.46	18.87	1.30	<0.0001

**P20701**	ITGAL	35	Isoform 2 of Integrin alpha-L	14.57	18.76	1.29	0.0002
**P11215**	ITGAM	13	Isoform 2 of Integrin alpha-M	11.17	22.17	1.99	<0.0001
**O60711**	LPXN	11	Isoform 2 of Leupaxin	7.50	25.84	3.45	<0.0001
**Q6WCQ1**	MPRIP	10	Isoform 2 of Myosin phosphatase Rho-interactin	18.43	14.90	-1.24	0.0012
**Q9UJF2**	RASAL2	29	Isoform 2 of Ras GTPase-activating pro	19.05	14.29	-1.33	<0.0001
**Q7LDG7**	RASGRP2	18	Isoform 2 of RAS guanyl-releasing protein 2	19.47	13.87	-1.40	<0.0001
**Q96RF0**	SNX18	17	Isoform 2 of Sorting nexin-18	19.31	14.02	-1.38	<0.0001
**Q9UMS6**	SYNPO2	2	Isoform 2 of Synaptopodin-2	24.15	9.18	-2.63	<0.0001
**O00468**	AGRN	28	Isoform 3 of Agrin	10.83	22.50	2.08	<0.0001
**P35611**	ADD1	19	Isoform 3 of Alpha-adducin	18.58	14.75	-1.26	0.0051
**Q16643**	DBN1	20	Isoform 3 of Drebrin	19.14	14.20	-1.35	<0.0001
**P06396**	GSN	197	Isoform 3 of Gelsolin	18.68	14.65	-1.27	<0.0001
**P33241**	LSP1	2	Isoform 3 of Lymphocyte-specific protein 1	23.57	9.76	-2.42	<0.0001
**Q13137**	CALCOCO2	13	Isoform 4 of Calcium-binding and coiled-coil domain-containing	19.83	13.50	-1.47	<0.0001
**Q8TDZ2**	MICAL1	80	Isoform 4 of Protein-methionine sulfoxide	17.98	15.35	-1.17	0.0092
**Q9P266**	KIAA1462	4	Junctional protein associated with coronary artery disease	19.53	13.80	-1.42	0.0249
**Q86UP2**	KTN1	96	Kinectin	17.87	15.47	-1.15	0.04
**Q9NQ48**	LZTFL1	17	Leucine zipper transcription	19.13	14.20	-1.35	0.0191

			factor-like protein 1				
**P07333**	CSF1R	2	Macrophage colony-stimulating factor 1 receptor	24.75	8.58	-2.88	0.0021
**Q9UPN3**	MACF1	152	Microtubule-actin cross-linking factor 1, isoforms 1/2/3/5	18.77	14.56	-1.29	<0.0001
**P46821**	MAP1B	59	Microtubule-associated protein 1B	11.40	21.93	1.92	<0.0001
**P29966**	MARCKS	10	Myristoylated alanine-rich C-kinase substrate	14.83	18.50	1.25	<0.0001
**O14745**	SLC9A3R1	17	Na(+)/H(+) exchange regulatory cofactor NHE-RF1	19.59	13.74	-1.43	<0.0001
**Q8NF91**	SYNE1	2	Nesprin-1	20.15	13.18	-1.53	0.0009
**Q09666**	AHNAK	420	Neuroblast differentiation-associated protein	15.46	17.87	1.16	0.0374
**P61601**	NCALD	21	Neurocalcin-delta	19.10	14.23	-1.34	<0.0001
**Q04721**	NOTCH2	18	Neurogenic locus notch homolog protein 2	15.30	18.04	1.18	0.0178
**Q0ZGT2**	NEXN	2	Nexilin	13.71	19.62	1.43	0.0111
**Q14980**	NUMA1	142	Nuclear mitotic apparatus protein 1	18.02	15.31	-1.18	0.001
**Q96RD7**	PANX1	3	Pannexin-1	12.65	20.68	1.63	<0.0001
**O75781**	PALM	8	Paralemmin-1	19.15	14.19	-1.35	0.0005
**Q8TCU6**	PREX1	55	Phosphatidylinositol 3,4,5-trisphosphate-dependent Rac exchanger 1 protein	19.33	14.00	-1.38	<0.0001
**Q6NYC8**	PPP1R18	14	Phostensin	14.48	18.85	1.30	0.0008
**Q15149**	PLEC	239	Plectin	13.84	19.49	1.41	<0.0001
**Q8N8S7**	ENAH	2	Protein enabled homolog	13.58	19.75	1.45	0.0139
**Q96ED9**	HOOK2	2	Protein Hook homolog 2	20.39	12.94	-1.58	0.0002

**Q05655**	PRKCD	57	Protein kinase C delta type	14.64	18.69	1.28	0.0001
**Q8TE77**	SSH3	24	Protein phosphatase Slingshot homolog 3	19.81	13.52	-1.46	<0.0001
**P26447**	S100A4	23	Protein S100-A4	18.26	15.07	-1.21	0.0279
**P25815**	S100P	4	Protein S100-P	8.99	24.35	2.71	<0.0001
**O75695**	RP2	19	Protein X	18.05	15.29	-1.18	<0.0001
**O15553**	MEFV	5	Pyrin	13.54	19.79	1.46	0.0044
**Q96QF0**	RAB3IP	3	Rab-3A-interacting protein	13.61	19.73	1.45	0.0093
**O15211**	RGL2	15	Ral guanine nucleotide dissociation stimulator-like 2	18.78	14.56	-1.29	0.0015
**Q9H6Z4**	RANBP3	16	Ran-binding protein 3	18.66	14.68	-1.27	0.0063
**O14807**	MRAS	3	Ras-related protein M-Ras	12.66	20.67	1.63	0.0059
**Q92930**	RAB8B	6	Ras-related protein Rab-8B	13.81	19.53	1.41	0.0092
**Q86X27**	RALGPS2	3	Ras-specific guanine nucleotide-releasing factor RalGPS2	19.63	13.70	-1.43	0.0161
**Q53QZ3**	ARHGAP15	18	Rho GTPase-activating protein 15	18.79	14.55	-1.29	0.0012
**Q68EM7**	ARHGAP17	23	Rho GTPase-activating protein 17	19.28	14.05	-1.37	0.0234
**Q969X0**	RILPL2	7	RILP-like protein 2	13.08	20.26	1.55	0.0048
**Q9GZR1**	SENP6	10	Sentrin-specific protease 6	19.62	13.71	-1.43	<0.0001
**Q96GX5**	MASTL	12	Serine/threonine-protein kinase greatwall	13.87	19.46	1.40	<0.0001
**Q8TDX7**	NEK7	13	Serine/threonine-protein kinase Nek7	18.48	14.85	-1.24	0.0017
**P02787**	TF	2	Serotransferrin	15.52	17.81	1.15	<0.0001
**P16949**	STMN1	49	Stathmin	18.94	14.40	-1.32	<0.0001

**Q9UQE7**	SMC3	98	Structural maintenance of chromosomes protein 3	17.90	15.44	-1.16	0.0274
**Q9UH65**	SWAP70	46	Switch-associated protein 70	18.58	14.75	-1.26	0.0003
**Q8N3V7**	SYNPO	2	Synaptopodin	20.44	12.89	-1.59	<0.0001
**O00560**	SDCBP	22	Syntenin-1	15.33	18.00	1.17	0.0241
**P36897**	TGFBR1	5	TGF-beta receptor type-1	12.43	20.90	1.68	0.0005
**O94759**	TRPM2	3	Transient receptor potential cation channel subfamily M member 2	12.33	21.00	1.70	<0.0001
**Q9NZR1**	TMOD2	7	Tropomodulin-2	13.46	19.88	1.48	0.0007
**P67936**	TPM4	26	Tropomyosin alpha-4 chain	13.16	20.17	1.53	<0.0001
**P23381**	WARS	56	Tryptophan--tRNA ligase, cytoplasmic	15.53	17.81	1.15	0.0109
**Q15814**	TBCC	21	Tubulin-specific chaperone C	18.42	14.91	-1.24	0.0092
**P21580**	TNFAIP3	17	Tumor necrosis factor alpha-induced protein 3	9.47	23.87	2.52	<0.0001
**P08631**	HCK	26	Tyrosine-protein kinase	13.19	20.14	1.53	<0.0001
**Q9NQC7**	CYLD	18	Ubiquitin carboxyl-terminal hydrolase	13.98	19.36	1.38	0.0003
**B0I1T2**	MYO1G	51	Unconventional myosin-Ig	14.26	19.08	1.34	0.0054
**Q92614**	MYO18A	72	Unconventional myosin-XVIIIa	18.74	14.59	-1.28	<0.0001
**Q03405**	PLAUR	9	Urokinase plasminogen activator surface receptor	11.02	22.31	2.03	<0.0001
**P08670**	VIM	58	Vimentin	8.95	24.38	2.72	<0.0001
**P04004**	VTN	6	Vitronectin	11.67	21.66	1.86	<0.0001

Table S2. Differentially expressed UPR proteins identified in PSP treated THP1-Blue-CD14 cells.

Accesion #	GENE	Quantified Spectra Counts	Protein Name	Average Expression in Control	Average Expression in PSP	Fold Change	Adjusted P Value
**O60613**	SELENOH	10	15 kDa selenoprotein H	19.43	13.90	-1.40	<0.0001
**P05067**	APP	23	Amyloid beta A4 protein	14.05	19.28	1.37	<0.0001
**P32929**	CTH	13	Cystathionine gamma-lyase	13.00	20.33	1.56	<0.0001
**Q9H3Z4**	DNAJC5	7	DnaJ homolog subfamily C member 5	17.90	15.44	-1.16	0.0287
**Q92611**	EDEM1	19	ER degradation-enhancing alpha-mannosidase-like protein 1	14.82	18.52	1.25	0.0278
**Q9NRD1**	FBXO6	4	F-box only protein 6	12.31	21.03	1.71	<0.0001
**Q06210**	GFPT1	62	Glutamine--fructose-6-phosphate aminotransferase [isomerizing] 1	14.87	18.47	1.24	0.0084
**Q9HAV7**	GRPEL1	13	GrpE protein homolog 1, mitochondrial	14.22	19.11	1.34	0.0003
**Q92598**	HSPH1	82	Heat shock protein 105 kDa	14.40	18.93	1.31	<0.0001
**P08238**	HSP90AB1	272	Heat shock protein HSP 90-beta	15.44	17.89	1.16	0.0299
**Q9Y4L1**	HYOU1	120	Hypoxia up-regulated protein 1	14.79	18.55	1.25	<0.0001
**Q96HD1**	CRELD1	9	Isoform 2 of Cysteine-rich with EGF-like domain protein 1	14.14	19.20	1.36	0.0373
**P04233**	CD74	14	Isoform 2 of HLA class II	12.98	20.35	1.57	<0.0001

			histocompatibility antigen ga				
**Q5F1R6**	DNAJC21	2	Isoform 3 of DnaJ homolog subfamily C member 21	19.58	13.75	-1.42	<0.0001
**Q14145**	KEAP1	10	Kelch-like ECH-associated protein 1	19.78	13.55	-1.46	<0.0001
**Q13451**	FKBP5	39	Peptidyl-prolyl cis-trans isomerase	14.00	19.33	1.38	<0.0001
**O00459**	PIK3R2	15	Phosphatidylinositol 3-kinase regulatory subunit beta	18.77	14.57	-1.29	0.0015
**P02545**	LMNA	111	Prelamin-A/C	14.46	18.88	1.31	<0.0001
**P07237**	P4HB	259	Protein disulfide-isomerase	15.35	17.99	1.17	0.0086
**Q9BZQ8**	FAM129A	44	Protein Niban	14.18	19.16	1.35	<0.0001
**Q58FG1**	HSP90AA4P	2	Putative heat shock protein HSP 90-alpha A4	18.42	14.92	-1.23	0.0244
**O75460**	ERN1	5	Serine/threonine-protein kinase/endoribonuclease IRE1	13.86	19.47	1.41	0.0359
**Q96EQ0**	SGTB	6	Small glutamine-rich tetratricopeptide repeat-containing protein beta	12.71	20.63	1.62	0.0012
**P38646**	HSPA9	225	Stress-70 protein, mitochondrial	15.13	18.20	1.20	0.0004
**Q8NBS9**	TXNDC5	26	Thioredoxin domain-containing protein 5	15.59	17.74	1.14	0.0017
**O14657**	TOR1B	8	Torsin-1B	12.03	21.30	1.77	<0.0001

**Q9NS93**	TM7SF3	3	Transmembrane 7 superfamily member 3	19.55	12.83	-1.52	0.0099
**Q96RU7**	TRIB3	5	Tribbles homolog 3	13.75	19.59	1.43	0.0015
